# A platinum(IV) prodrug strategy to overcome glutathione-based oxaliplatin resistance

**DOI:** 10.1038/s42004-022-00661-z

**Published:** 2022-04-06

**Authors:** Philipp Fronik, Michael Gutmann, Petra Vician, Mirjana Stojanovic, Alexander Kastner, Petra Heffeter, Christine Pirker, Bernhard K. Keppler, Walter Berger, Christian R. Kowol

**Affiliations:** 1 University of Vienna, Faculty of Chemistry, Institute of Inorganic Chemistry, Waehringer Strasse 42, 1090 Vienna, Austria; 2 Center of Cancer Research and Comprehensive Cancer Center, Medical University of Vienna, Borschkegasse 8a, 1090 Vienna, Austria; 3 Research Cluster “Translational Cancer Therapy Research”, 1090 Vienna, Austria

**Keywords:** Drug delivery, Drug discovery and development

## Abstract

Clinical efficacy of oxaliplatin is frequently limited by severe adverse effects and therapy resistance. Acquired insensitivity to oxaliplatin is, at least in part, associated with elevated levels of glutathione (GSH). In this study we report on an oxaliplatin-based platinum(IV) prodrug, which releases *L*-buthionine-*S*,*R*-sulfoximine (BSO), an inhibitor of glutamate-cysteine ligase, the rate-limiting enzyme in GSH biosynthesis. Two complexes bearing either acetate (**BSO-OxOAc**) or an albumin-binding maleimide (**BSO-OxMal**) as second axial ligand were synthesized and characterized. The in vitro anticancer activity of **BSO-OxOAc** was massively reduced in comparison to oxaliplatin, proving its prodrug nature. Nevertheless, the markedly lower intracellular oxaliplatin uptake in resistant HCT116/OxR cells was widely overcome by **BSO-OxOAc** resulting in distinctly reduced resistance levels. Platinum accumulation in organs of a colorectal cancer mouse model revealed higher tumor selectivity of **BSO-OxMal** as compared to oxaliplatin. This corresponded with increased antitumor activity, resulting in significantly enhanced overall survival. **BSO-OxMal**-treated tumors exhibited reduced GSH levels, proliferative activity and enhanced DNA damage (pH2AX) compared to oxaliplatin. Conversely, pH2AX staining especially in kidney cells was distinctly increased by oxaliplatin but not by **BSO-OxMal**. Taken together, our data provide compelling evidence for enhanced tumor specificity of the oxaliplatin(IV)/BSO prodrug.

## Abstract (web summary)

Platinum(IV) complexes have the potential to overcome several limitations and reduce adverse effects of platinum-based cancer therapy. Resistance to clinically approved platinum(II) drugs is, at least in part, associated with elevated levels of glutathione. Here, the authors report on an oxaliplatin-based platinum(IV) prodrug, which releases *L*-buthionine-*S*,*R*-sulfoximine, an inhibitor of the rate-limiting enzyme in glutathione biosynthesis, to circumvent glutathione-based resistance mechanisms.

## Introduction

With over 18 million new cases and over 9 million fatalities in 2018, cancer is still the second leading cause of death worldwide^1,2^. Besides radiation therapy and surgery, chemotherapy is one of the main choices for treatment. Among the latter, platinum(II) complexes still have a large field of application, even though their history dates back to the discovery of cisplatin in the 1960’s^3^. Cisplatin and the other two worldwide approved platinum(II) complexes, carboplatin, and oxaliplatin, are used for a wide spectrum of cancer types, such as lung, testicular, prostate, ovarian, colon, and head-and-neck carcinoma^4^. Especially combination therapies with modern immunotherapeutics show massive synergism and are therefore already approved e.g. as first-line therapy against lung cancer^5,6^. The use of platinum(II) complexes, however, frequently leads to hair loss, nausea, and other more severe side effects, e.g., oto-, neuro- and nephrotoxicity, which often can be dose-limiting^4^. In addition, many patients are either intrinsically resistant or develop chemoresistance throughout the therapy, which can lead to tumor relapse after an initial response^7,8^. Platinum(IV) complexes have the potential to overcome at least some of the drawbacks of the currently approved platinum(II) drugs. They are significantly more inert and, thus, exert much lower toxic effects on a systemic level. In addition, the complexes can be reduced to their active platinum(II) species in the reductive/hypoxic environment of the malignant tissue, leading to a more tumor-specific activation. Different platinum(IV) complexes have been assessed in clinical trials, but none of them has been approved yet^9^. Satraplatin, the most promising platinum(IV) drug candidate to date, failed in a phase III clinical trial, likely because it suffered from fast reduction already in red blood cells^10,11^. Thus, one way to potentially improve this class of compounds, is by enhancing their plasma half-life and tumor selectivity, e.g. by passive targeting via albumin^12,13^. We have previously reported on an oxaliplatin-based platinum(IV) complex featuring a maleimide moiety which has shown high antitumor activity in vivo^14^. The maleimide of the prodrug rapidly and selectively reacts with the only free thiol group of albumin at position 34. The covalently bound drug is then transported to the tumor tissue, where it accumulates via two mechanisms: 1) cancer cells have been demonstrated to use albumin as an amino acid source and, thus, show an increased uptake of this plasma protein^15,16^, and 2) the enhanced permeability and retention (EPR) effect, which enables macromolecules to enrich in cancerous tissue, due to disordered and thus fenestrated vascularization and impaired lymphatic drainage^17^.

A further option for improvement is the introduction of additional bioactive moieties into one or both axial positions of the platinum(IV) complex, which are released upon reduction. In this way, synergistic agents can be administered selectively to the site of reduction-mediated platinum(II) release. This approach has already been successfully investigated and, just recently, attachment of as many as three different active moieties to a platinum core has been reported^18,19^. Bioactive ligands might for example be chosen to specifically block acquired resistance mechanisms. Multiple reports suggest elevated glutathione (GSH) levels in cancer cells or extracellularly in the tumor microenvironment via the release of biothiols (GSH, cysteine) e.g. by cancer-associated fibroblasts^20,21^. Both, passive as well as glutathione-S-transferase (GST)-catalyzed adduct formation may inactivate platinum(II) drugs (including oxaliplatin) and/or facilitate their efflux^22–25^. Promising data on platinum(IV) prodrugs of cisplatin with the GST enzymes inhibitor ethacrynic acid as axial ligand have been reported^26–28^. Such approaches are missing for oxaliplatin so far. A more comprehensive strategy to avoid GSH-based platinum drug resistance mechanisms is based on suppression of GSH synthesis in the malignant tissue, which would—besides GST-catalyzed drug conjugation in cancer cells—also target extracellular oxaliplatin inactivation via GSH adduct formation^20^. *L*-Buthionine-*S*,*R*-sulfoximine (BSO) is a potent, specific and irreversible inhibitor of glutamate-cysteine ligase (GCL), the rate-limiting enzyme in GSH biosynthesis^29,30^. BSO has shown the ability to re-sensitize doxorubicin- and cisplatin-resistant cancer cells to drug treatment and has demonstrated outstanding synergism with platinum drugs in a murine ovarian carcinoma model in vivo^31–33^. Furthermore, BSO has remarkable antitumor activity as a sensitizing agent in combination with the alkylating agent melphalan as well as arsenic trioxide and has even advanced to clinical trials^34–38^. However, BSO suffers from fast metabolism and excretion, as a pharmacokinetic study showed a plasma half-life of <2 h in patients^39^. Therefore, continuous 72 h infusions had to be applied in order to reach the desired effect regarding GSH depletion^30,40^. Consequently, we hypothesized that attachment of BSO to a platinum(IV) prodrug has the potential to improve its pharmacokinetics and tumor specificity, especially when using an albumin-binding maleimide moiety, which is well-known to increase both, plasma half-life and cancer targeting^14^. Additionally, this approach should allow spatially and temporally controlled, simultaneous release of the two synergistic compounds. After activation by reduction, the released BSO should decrease the GSH levels especially in malignant cells, reducing inactivation of oxaliplatin via formation of platinum-GSH adducts and their cellular excretion via efflux pumps of the ABCC family^41,42^. Consequently, the aim of this study was to synthesize the first BSO-platinum(IV) prodrugs, both with and without an albumin-targeting maleimide moiety, and to assess their antitumor activity in vitro and in vivo. Herein we demonstrate, that the respective albumin-targeting prodrug not only possesses prolonged serum-half-life and the ability to lower intratumoral GSH levels stronger as compared to oxaliplatin, but also improves overall survival in an in vivo mouse model, combined with an advantageous toxicity profile.

## Results and discussion

### Chemical synthesis

**Fig. 1:**
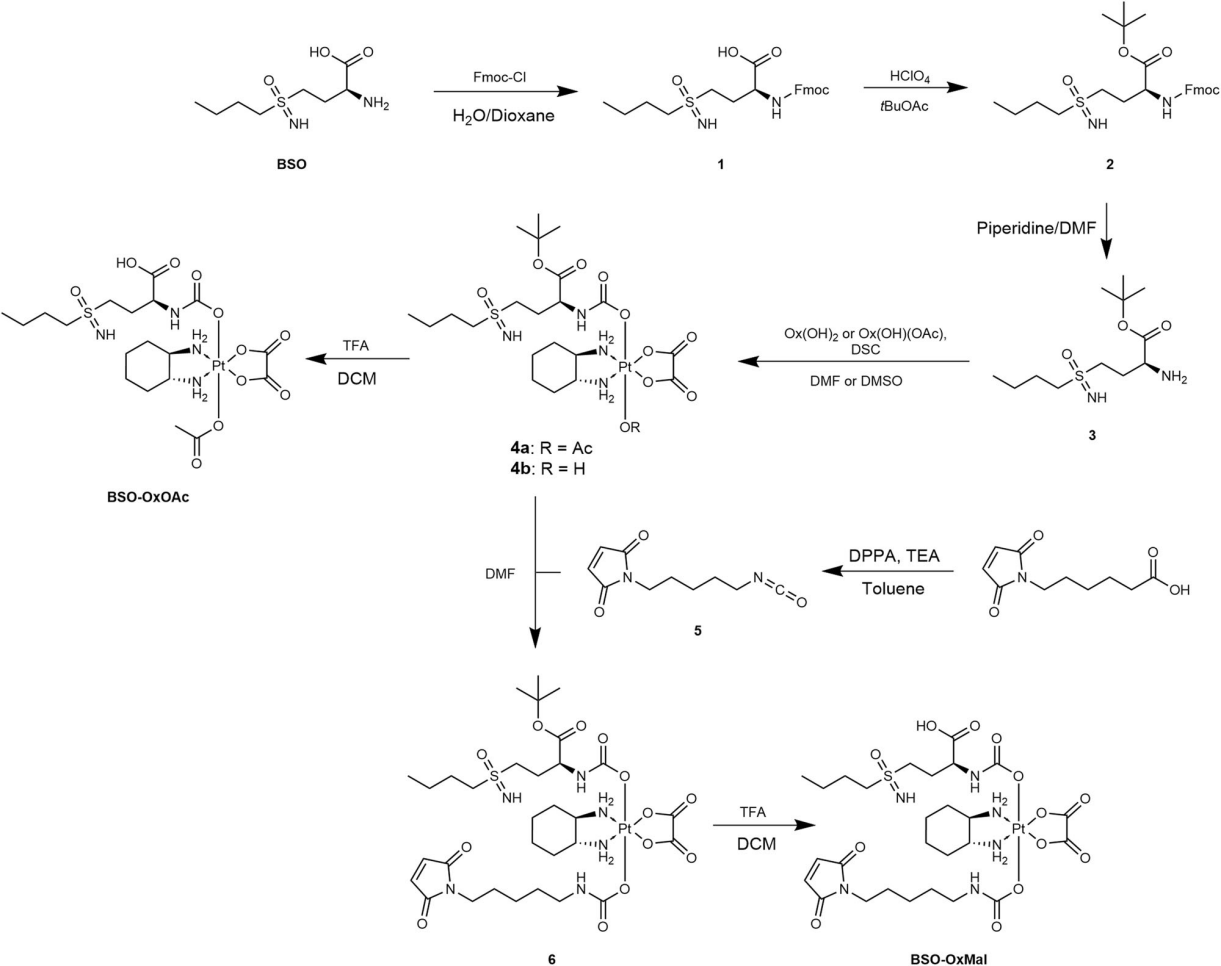
Synthetic route to platinum(IV) prodrugs **BSO-OxOAc** and **BSO-OxMal**. The respective experimental conditions can be found in the Methods Section.

Since the very recent development of 1,1’-disuccinimidyl carbonate-mediated coupling of amine-containing bioactive molecules to platinum(IV) cores by Gibson *et al*., the preparation of carbamato-ligands has become easy and safe in comparison to methods using phosgene or its derivatives^43^. However, in case of BSO, the low solubility of the parent compound in organic solvents (including dimethylsulfoxide (DMSO)) and the presence of interfering functional groups, such as the sulfoximine and the carboxylic acid, prohibited a straightforward synthesis. Using a transient Fmoc-protected species (Fig. 1), a BSO-*t*-butyl ester (**3**) was synthesized which then could be successfully coupled to the hydroxido ligand of the oxaliplatin(IV) precursor **OxOH/OAc** to obtain complex **4b**. Deprotection with trifluoroacetic acid (TFA) in dichloromethane yielded **BSO-OxOAc** which was again purified by preparative high-performance liquid chromatography (HPLC). For the synthesis of **BSO-OxMal**, the **Ox(OH)**_**2**_ precursor was used and, after the addition of one BSO ligand, maleimide isocyanate (**5**) was coupled to the remaining hydroxido group of the platinum center. Acidic deprotection and preparative HPLC purification generated the platinum(IV) prodrug **BSO-OxMal**. The obtained complexes were characterized by ^1^H-and ^13^C-NMR spectroscopy, mass spectrometry, and elemental analysis. Notably, several doublets with almost identical chemical shifts could be observed in the respective ^13^C-NMR spectra of the platinum(IV) prodrugs (see Methods Section and Supplementary Figs. 1 and 2). This can be explained by the presence of a diastereomeric mixture of the commercially available *L*-buthionine-*S*, *R*-sulfoximine precursor. The compound is stereochemically pure at the α-carbon of the amino acid, but a *S*, *R* mixture at the sulfur-atom of the chiral sulfoximine group. This results in a splitting of some ^13^C-NMR signals. However, no differences in retention time in the HPLC chromatograms were visible and, consequently, no separation during the HPLC purification could be performed. Of note, also in the clinical studies of BSO the diastereomeric mixture was used^30,38^.

### Complex stability

**Fig. 2:**
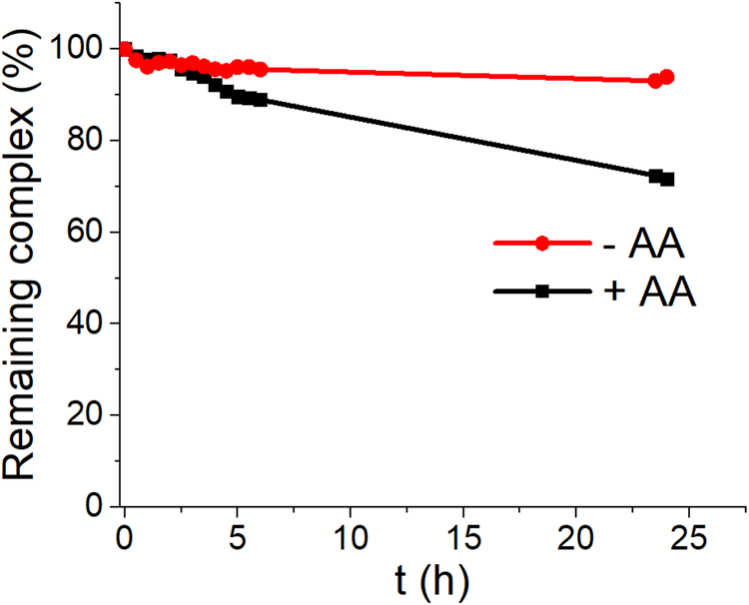
UHPLC stability kinetic measurements of **BSO-OxOAc**. The stability of the complex (1 mM) was measured in 100 mM phosphate buffer (pH 7.4; 20 °C) and the reduction in the presence of 10 mM AA in 500 mM phosphate buffer (pH 7.4; 20 °C).

As the synthesized platinum(IV) prodrugs need to be delivered to the tumor site before being activated, their stability in aqueous solution as well as their resistance to endogenous reductants is of utmost importance. However, since maleimides are prone to slow hydrolysis at pH-values >6, the acetato-derivative **BSO-OxOAc** was used to investigate these properties. The aqueous stability of **BSO-OxOAc** in phosphate buffer (pH 7.4), as well as its reduction kinetics with a 10-fold excess of ascorbic acid (AA) were measured by ultra-high-performance liquid chromatography (UHPLC). The complex revealed high aqueous stability with a calculated half-life of ~240 h, and slow reduction kinetics (t_½_ ~51 h; Fig. 2) in line with analogous oxaliplatin-based platinum(IV) prodrugs (representative UHPLC chromatograms can be found in Supplementary Fig. 3)^14^.

### Albumin binding

**Fig. 3:**
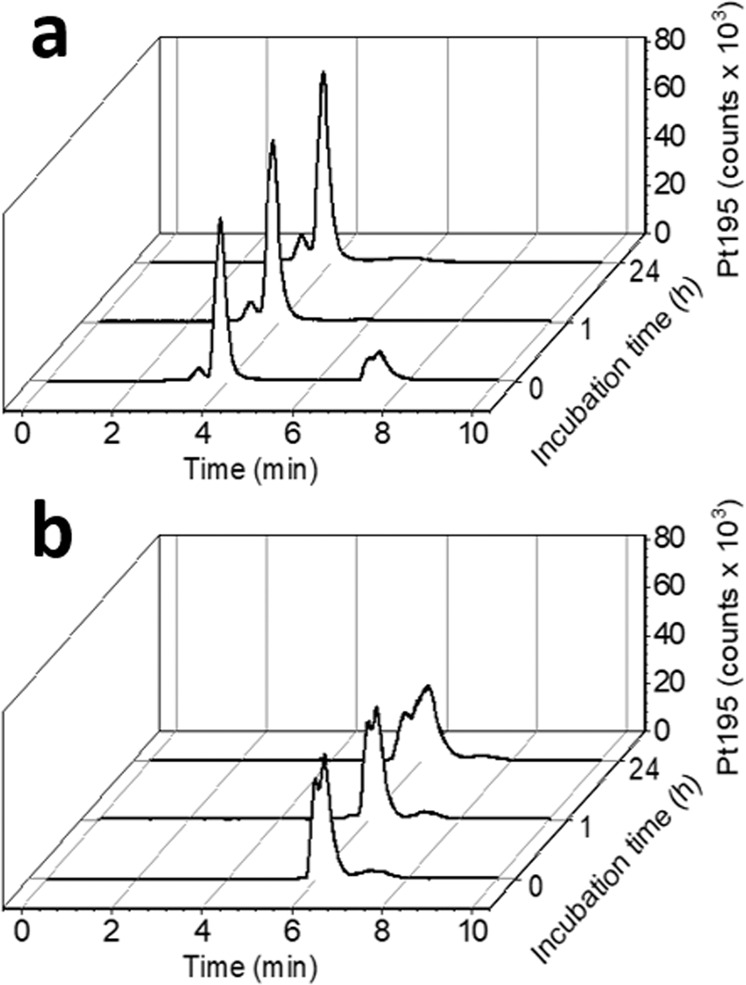
Platinum-SEC-ICP-MS traces of **BSO-OxMal** and **BSO-OxOAc**. (**a**) shows **BSO-OxMal** and (**b**) **BSO-OxOAc**. The compounds were incubated in fetal calf serum at 37 °C and measured after 0, 1, and 24 h. The peaks at t_r_ > 6 min represent low molecular weight platinum, the peak at t_r_ ~4 min albumin-bound platinum. The quantitative analysis of the albumin binding is summarized in Supplementary Table 1.

In order to prove the albumin-binding ability of **BSO-OxMal**, we incubated both drugs, **BSO-OxMal** and **BSO-OxOAc**, in fetal calf serum at 37 °C and measured the platinum signal by size exclusion chromatography inductively coupled plasma mass spectrometry (SEC-ICP-MS). As depicted in Fig. 3a, in case of **BSO-OxMal**, already after one hour all platinum was bound to the high molecular weight fraction at the expected retention time of albumin (~4.0 min; at 3.4 min the albumin-dimer is visible), demonstrating a rapid and selective attachment to the biomolecule (the respective sulfur trace of serum can be found in Supplementary Fig. 4, the quantitative analysis in Supplementary Table 1). The fact that no **BSO-OxMal** is left in the low molecular weight fraction indicates that the hydrolysis of the maleimide is negligible under these conditions. In addition, no significant changes could be observed after 24 h indicating a high stability of the albumin conjugate in serum. In contrast, **BSO-OxOAc**, as expected, did not bind to albumin and all platinum remained in the low molecular weight fraction (t_r_ > 6 min) throughout 24 h (Fig. 3b).

### Anticancer activity in vitro and impact of platinum drug resistance

**Fig. 4:**
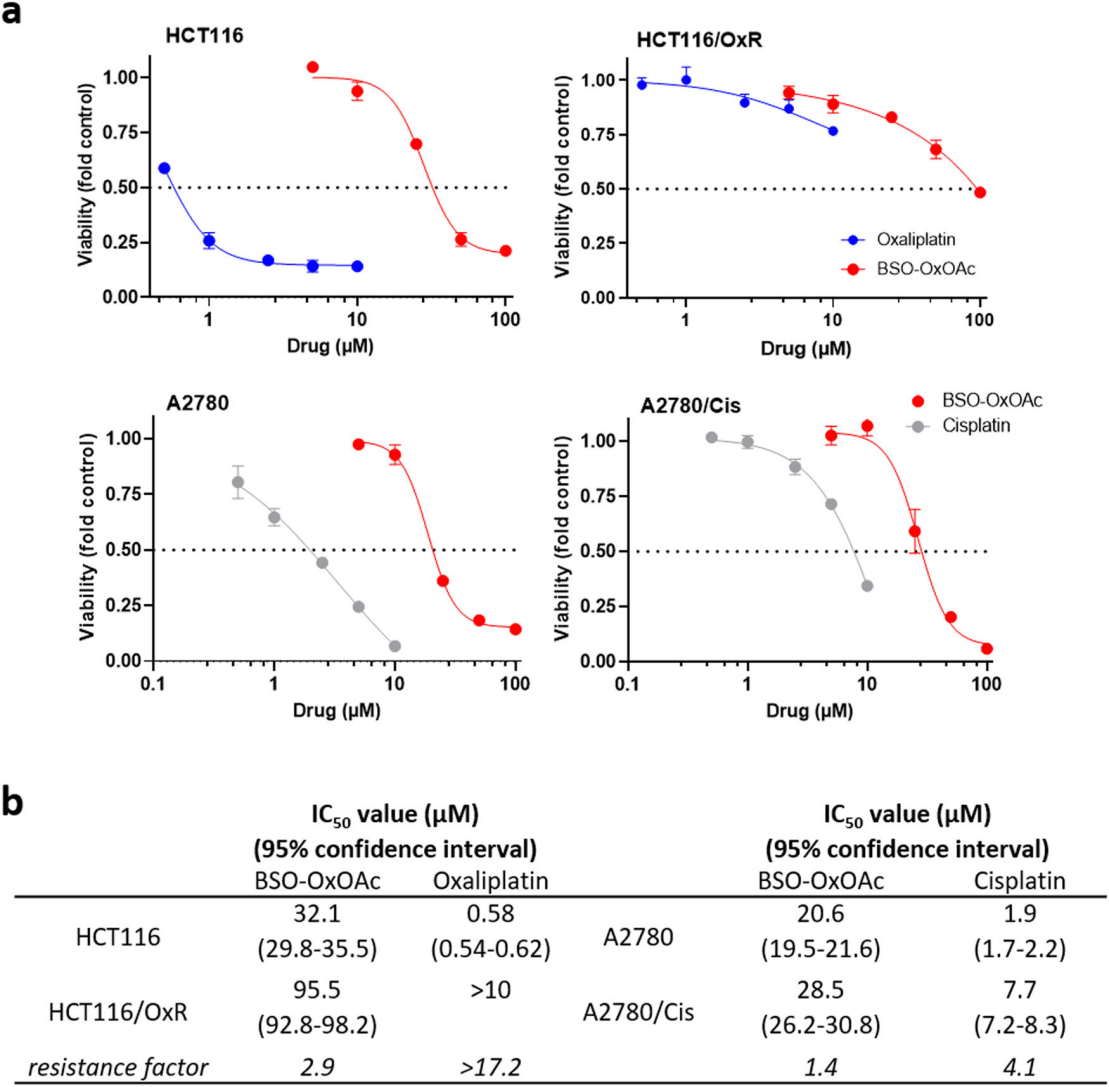
Impact of **BSO-OxOAc** as compared to oxaliplatin and cisplatin on the viability of the indicated cancer cell lines and their sublines with the respective acquired platinum drug resistance. **a** Impact of a 72 h continuous drug exposure on the viability of human colon cancer cells HCT116 and a oxaliplatin-resistant subline HCT116/OxR, as well as the human ovarian cancer cell model A2780 and the cisplatin-resistant subline A2780/Cis. One representative experiment out of at least three performed in triplicate is shown. Data points are depicted as mean ± SD. **b** IC_50_ values were derived from the dose-response curves as shown under (**a**) using the four-parameter logistic nonlinear regression model. Resistance factors were calculated by dividing the IC_50_ values for the resistant subline by those of the respective sensitive parental cell model.

In a first approach, the anticancer activity of the prodrug system was compared in cell culture in 72 h continuous drug exposure assays in the human colon cancer model HCT116 and the ovarian cancer model A2780, together with their respective sublines harboring acquired resistance against oxaliplatin (HCT116/OxR) and cisplatin (A2780/Cis). Determination of the anticancer activity of albumin-targeting maleimide drugs is limited to the in vivo situation, as components of the cell culture medium, such as e.g. free cysteine residues, are capable of reacting with the maleimide moiety. Hence, we subjected only the acetate-bearing **BSO-OxOAc** to in vitro cytotoxicity and drug accumulation testing. Confirming functionality of the prodrug concept, the in vitro cytotoxicity of **BSO-OxOAc** was massively reduced as compared to oxaliplatin in all malignant cell models (Fig. 4a), indicated by about 10- to 50-fold enhanced IC_50_ values (Fig. 4b). A comparably reduced cytotoxicity was also observed in the murine colon cancer model CT26 with a > 58-fold higher IC_50_ value (compare Supplementary Fig. 7). This reveals only limited platinum(IV) complex reduction and ligand release even during 72 h drug exposure. Nevertheless, when comparing the activity of **BSO-OxOAc** with that of oxaliplatin and cisplatin in the respective resistance models, the resistance factors for the platinum(IV) prodrug were markedly lower, indicating that the prodrug is distinctly less affected by the involved resistance mechanisms. Concerning non-malignant cell counterparts, oxaliplatin was distinctly active against keratinocytes (HaCat, IC_50_ 3.4 µM) and to a lesser extent against endothelial cells (BEC, IC_50_ 10.3 µM), while **BSO-OxOAc** was widely inactive against all non-malignant cell types tested (Supplementary Fig. 5). This suggests, as expected, an enhanced therapeutic window in case of the prodrug system.

**Fig. 5:**
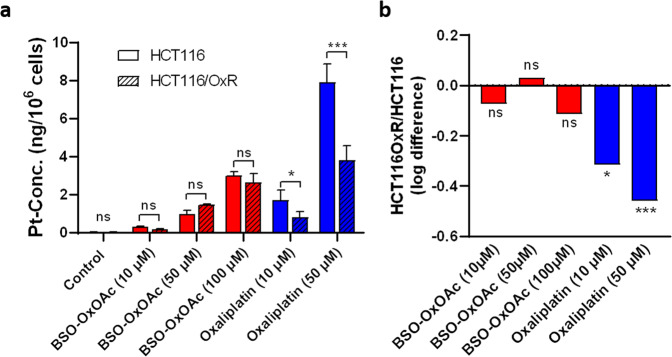
Cellular uptake of **BSO-OxOAc** as compared to free oxaliplatin and impact of acquired oxaliplatin resistance. **a** Parental HCT116 and oxaliplatin-resistant HCT116/OxR cells were exposed to the indicated concentrations of **BSO-OxOAc** and oxaliplatin for 3 h and cellular uptake quantified by ICP-MS. Data are depicted as mean ± SD. **b** Log-fold difference in the platinum accumulation between parental HCT116 and HCT116/OxR calculated from the data shown in (**a**). Statistical significance was tested using one-way ANOVA. *p < 0.05; ***p < 0.001.

In order to test whether changes in the intracellular drug accumulation play a critical role in the observed efficacy differences, we determined the cellular platinum content following exposure to **BSO-OxOAc** or oxaliplatin in HCT116 and HCT116/OxR cells by ICP-MS (Fig. 5a). In agreement with previous studies on other oxaliplatin-based platinum(IV) prodrug systems^44^, the overall cellular accumulation of **BSO-OxOAc** was distinctly lower than of oxaliplatin in both cell models (Fig. 5a). This suggests, that not only the reduced reactivity, but also the limited cellular accumulation of the platinum(IV) as compared to the platinum(II) complex explain the attenuated cytotoxic activity of **BSO-OxOAc**. Concerning oxaliplatin resistance, in support of previous results^45^, the intracellular accumulation of oxaliplatin was significantly reduced in HCT116/OxR cells as compared to the parental cell line. This confirms that the acquired platinum resistance phenotype is at least partly based on reduced uptake or enhanced export of the drug. In contrast, the accumulation of our novel prodrug **BSO-OxOAc** was only non-significantly altered in the parental vs. oxaliplatin-resistant HCT116 cells (Fig. 5). This corresponds well with the cytotoxicity data and points towards substantial circumvention of transport-mediated, acquired oxaliplatin resistance by our prodrug system. We have previously shown that the platinum accumulation differences between parental and resistant HCT116 cells increased over time^45^, strongly pointing towards a central impact of oxaliplatin efflux mechanisms, like GSH-conjugate transporters of the ABCC family^29,30,42^.

In case of the platinum(IV) prodrug system, intracellular activation by reduction should cooperate with extracellular reduction, the latter facilitating more efficient uptake of the released platinum(II) species. Therefore, short-term and long-term combination experiments of **BSO-OxOAc** with the reducing agent AA were performed. In the 72 h exposure assay, AA dose-dependently but only moderately enhanced the cytotoxic activity of the platinum(IV) complex in parental HCT116 cells, with clear-cut activation only at 250 µM. In contrast, already 25 µM AA significantly enhanced **BSO-OxOAc** cytotoxicity in the resistant subline (Supplementary Fig. 6) while, surprisingly, 250 µM AA alone was toxic in this cell model (data not shown) and, hence, could not be evaluated in the combination setting. The stronger impact of AA in the resistant subline supports the assumption that GSH is involved in the resistance phenotype, causing a stronger synergism of the released oxaliplatin and BSO. Accordingly, we have shown previously that HCT116/OxR cells are characterized by an enhanced GSH metabolism, supporting the resistance phenotype^45^. This activating impact of AA on **BSO-OxOAc** was even more pronounced in case of the murine colorectal carcinoma cell line CT26 (Supplementary Fig. 7).

Additionally, combination experiments with the reductive (bio)thiols *N*-acetylcysteine (NAC), cysteine, and GSH were performed, the two former also being GSH precursors. It has been shown that oxaliplatin, like other platinum compounds, spontaneously interacts with thiols under cell-free and *in-cellulo* conditions^25^. Co-exposure with NAC, cysteine, or GSH distinctly protected HCT116 cells from oxaliplatin cytotoxicity, with GSH having the strongest effect and cysteine the weakest (Supplementary Fig. 6b, c). In case of **BSO-OxOAc**, again a rather protective effect was detectable which was, however, distinctly weaker as compared to free oxaliplatin and without a clear-cut difference between the three reductive agents (Supplementary Fig. 6c). Together, these data suggest that free oxaliplatin is strongly inactivated by biothiols, probably with dominant extracellular conjugate formation, while **BSO-OxOAc** is protected against extracellular inactivation based on its prodrug nature.

**Fig. 6:**
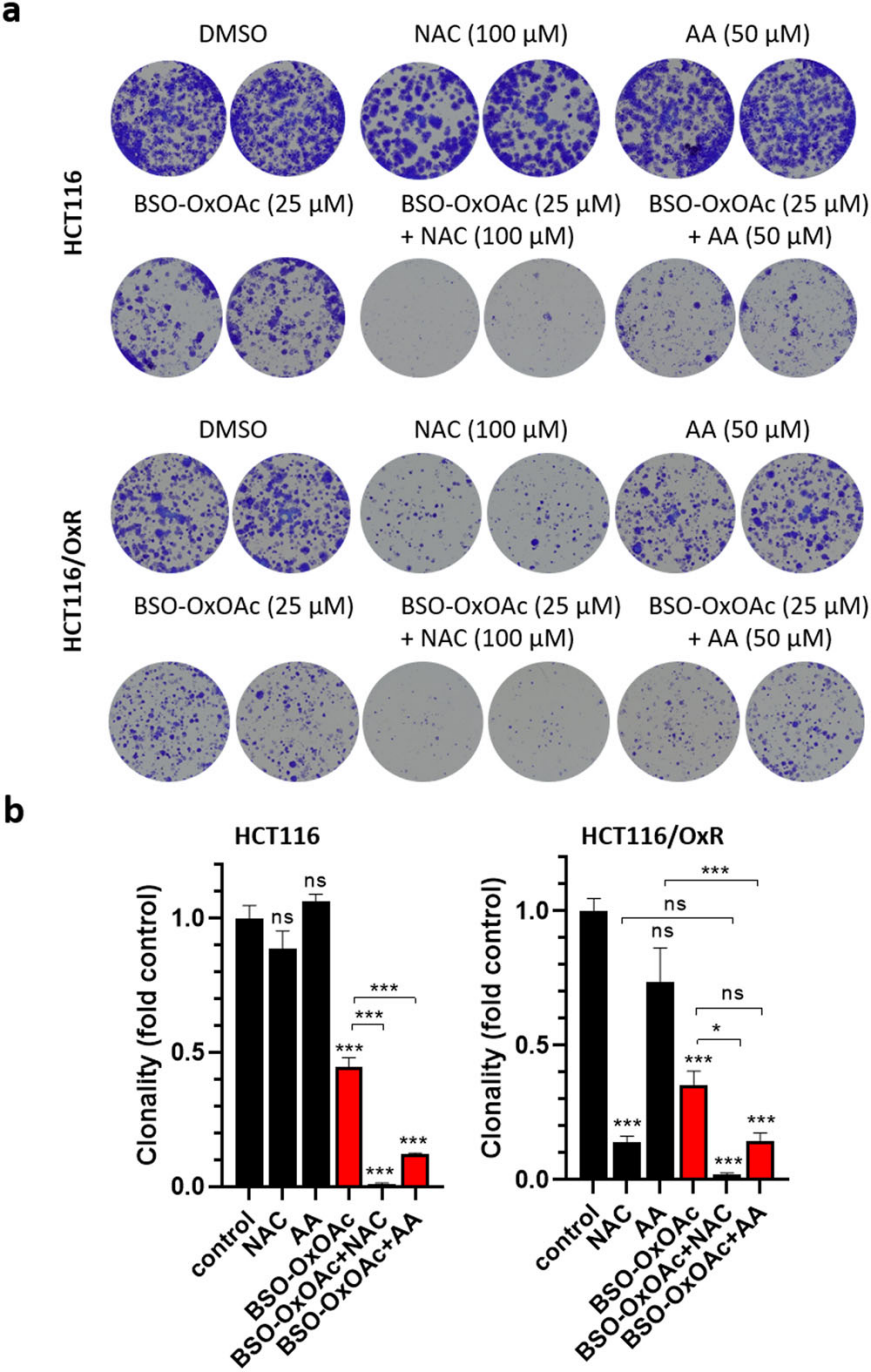
Impact of **BSO-OxOAc** as single agent and in combination with the reducing agents NAC (100 µM) and AA (50 µM) on the clonogenic potential of HCT116 cells and the subline with acquired oxaliplatin resistance (HCT116/OxR). Sparsely seeded cells (1 × 10^3^/24-well plate well) were exposed for 10 days to the indicated compounds and derived cell clones stained with crystal violet, photographed, and results evaluated by ImageJ software as described in the Methods section. One respective experiment out of three performed in duplicate is shown under (**a**) and the respective evaluation under (**b**), depicted as mean ± SD. Statistical significance was tested using two-way ANOVA. In all cases: *p < 0.05; **p < 0.01; ***p < 0.001.

Nevertheless, the anticancer activity of **BSO-OxOAc** in the presence of any tested reducing agent was still by far lower as compared to oxaliplatin, suggesting only limited reduction and platinum(II) release in this setting. Therefore, 10 day clonogenicity assays were performed. Under these experimental conditions, both AA and NAC markedly increased the anticancer activity of **BSO-OxOAc** (Fig. 6, Supplementary Fig. 9), suggesting continuous activation by the reducing agents over time, and, in case of NAC, additional GSH depletion by the released BSO ligand. In contrast to **BSO-OxOAc**, the activity of oxaliplatin was not influenced by AA (Supplementary Figs. 8 and 9). Also in the clonogenic setting, HCT116/OxR cells, distinctly resistant against oxaliplatin, again were per se hypersensitive against NAC and at higher concentrations also AA (Fig. 6). This supports complex redox balance deregulation in this resistance model. The enhanced GSH metabolism of HCT116/OxR cells, found in a previous metabolomics study mentioned above^45^, was also supported by our biochemical GSH data (Supplementary Fig. 10). While HCT116/OxR cells exhibited a moderately reduced basal GSH content as compared to parental cells, the GSH-reducing impact of BSO was distinctly stronger in the resistant subline. Whereas oxaliplatin did not alter the GSH content in this in vitro setting, **BSO-OxOAc** alone moderately, but in combination with AA distinctly reduced the cellular GSH content. The impact especially in the combined setting was again clearly stronger in the oxaliplatin-resistant subline, supporting enhanced GSH turnover as a determinant of the resistance phenotype.

### Serum pharmacokinetics and organ distribution

These promising in vitro results prompted us to investigate the biodistribution and anticancer activity in vivo against a colon cancer mouse model. As outlined in the introduction, we recently demonstrated that passive targeting to albumin via a maleimide moiety can distinctly enhance the tumor selectivity of platinum(IV) prodrugs^12–14^. Consequently, we favored **BSO-OxMal** for the in vivo experiments. In a first set of experiments, serum pharmacokinetics following a single, equimolar dose of **BSO-OxMal** as compared to oxaliplatin was determined for a 2-week time-frame by monitoring the platinum content in serum from facial vein blood using ICP-MS (Supplementary Fig. 11). The area under the concentration-time curve (AUC) for **BSO-OxMal** was drastically increased (>12-fold total area) and the serum half-life, depending on the regression model used, more than 10-fold higher as compared to oxaliplatin. Accordingly, serum concentrations were significantly higher for the prodrug at all time-points until 48 h post application. Together, these data demonstrate the increased exposure to the prodrug in the circulation due to albumin binding.

**Fig. 7:**
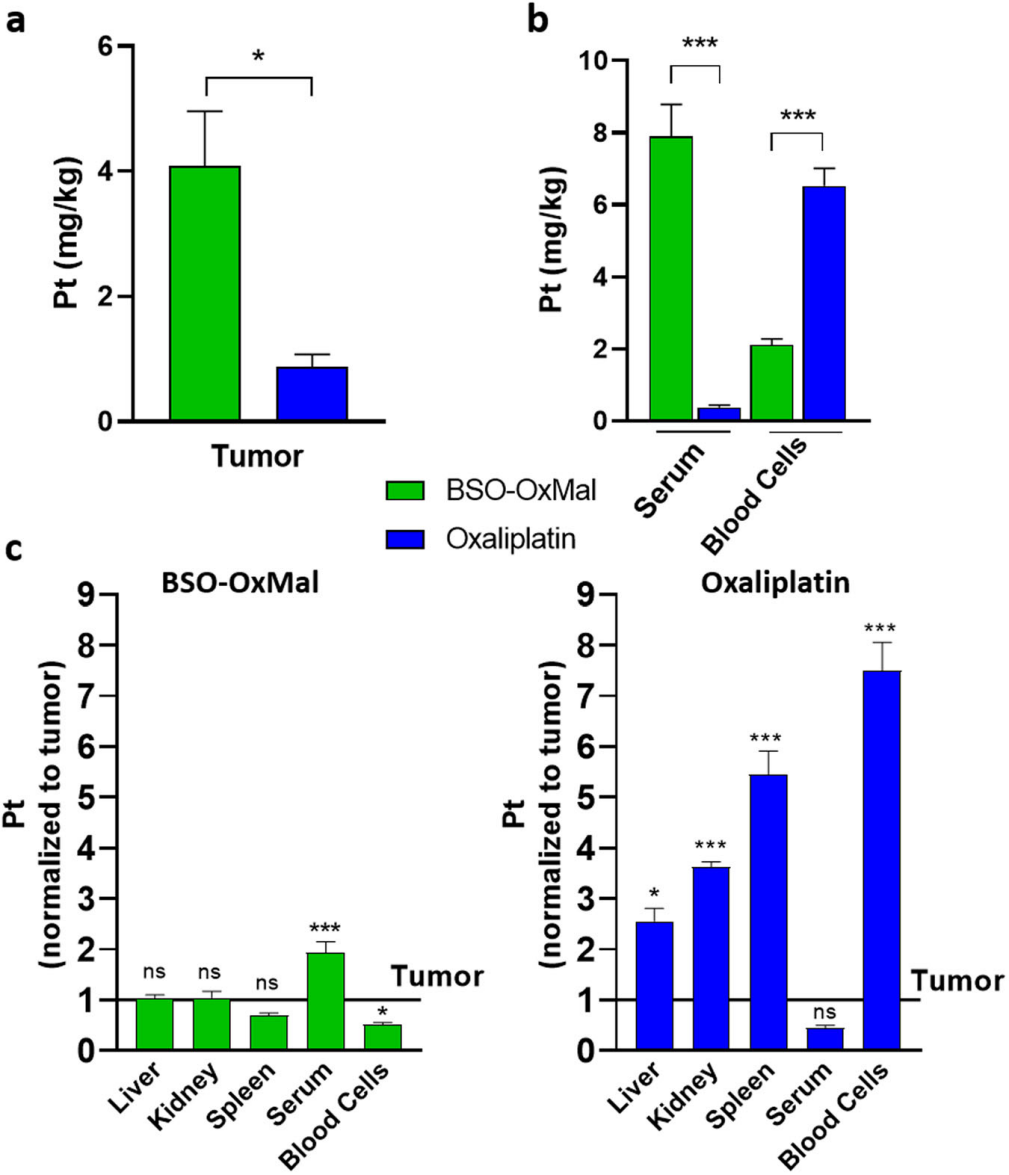
Tissue/organ distribution of **BSO-OxMal** compared to free oxaliplatin. Mice bearing CT26 allografts (*n* = 4 per group) were treated twice a week with equimolar concentrations of **BSO-OxMal** (23.5 mg/kg) or oxaliplatin (9 mg/kg) and sacrificed 24 h after the last drug dosing. Platinum contents of tumor tissue (**a**) and serum as compared to blood cells (**b**) was determined by ICP-MS. Statistical differences were tested by Student´s *t*-test. (**c**) Platinum levels in the indicated organs are given relative to the ones in the tumor tissue. Data are depicted as mean ± SD. Statistical significance was tested for all tissues/organs as compared to platinum levels in tumor tissue using one-way ANOVA. In all cases: *p < 0.05; ***p < 0.001.

Hence, we decided to analyze the anticancer activity of this compounds in vivo and determine organ distribution in a therapeutic setting. The contribution of the immune system to the anticancer activity of oxaliplatin is well-known^46,47^. In more detail, mice lacking T cells are widely unresponsive to the therapy, hence, adaptive immune responses in addition to oxaliplatin-induced DNA platination and DNA strand breaks are essential for the anticancer activity. Consequently, the oxaliplatin derivative was investigated in immune-competent murine allograft tumors. We determined the organ distribution of **BSO-OxMal** (after 1-week i.v. treatment with two doses; 24 h after second dosing) as compared to free oxaliplatin in the syngeneic colon adenocarcinoma CT26 mouse model. As compared to oxaliplatin, **BSO-OxMal** demonstrated significantly elevated accumulation in the tumor tissue (Fig. 7a). In agreement with the serum pharmacokinetics data (compare Supplementary Fig. 11), the albumin-targeted prodrug exhibited more than 20-fold increased serum platinum levels in comparison to oxaliplatin. In contrast, the uptake in blood cells was distinctly lower than in oxaliplatin-treated animals (Fig. 7b). The tumor selectivity of our prodrug was determined by comparing platinum organ/tumor ratios (Fig. 7c, d). This revealed similar (liver, kidney, spleen) or even reduced (blood cells) platinum levels in healthy as compared to malignant tissue in case of **BSO-OxMal**. In contrast, oxaliplatin exhibited significantly increased accumulation in all tested non-tumor versus tumor tissues. Thus, the more tumor-specific accumulation and activation of the platinum(IV) prodrug might help to limit adverse effects associated with oxaliplatin treatment.

### Anticancer activity in vivo

**Fig. 8:**
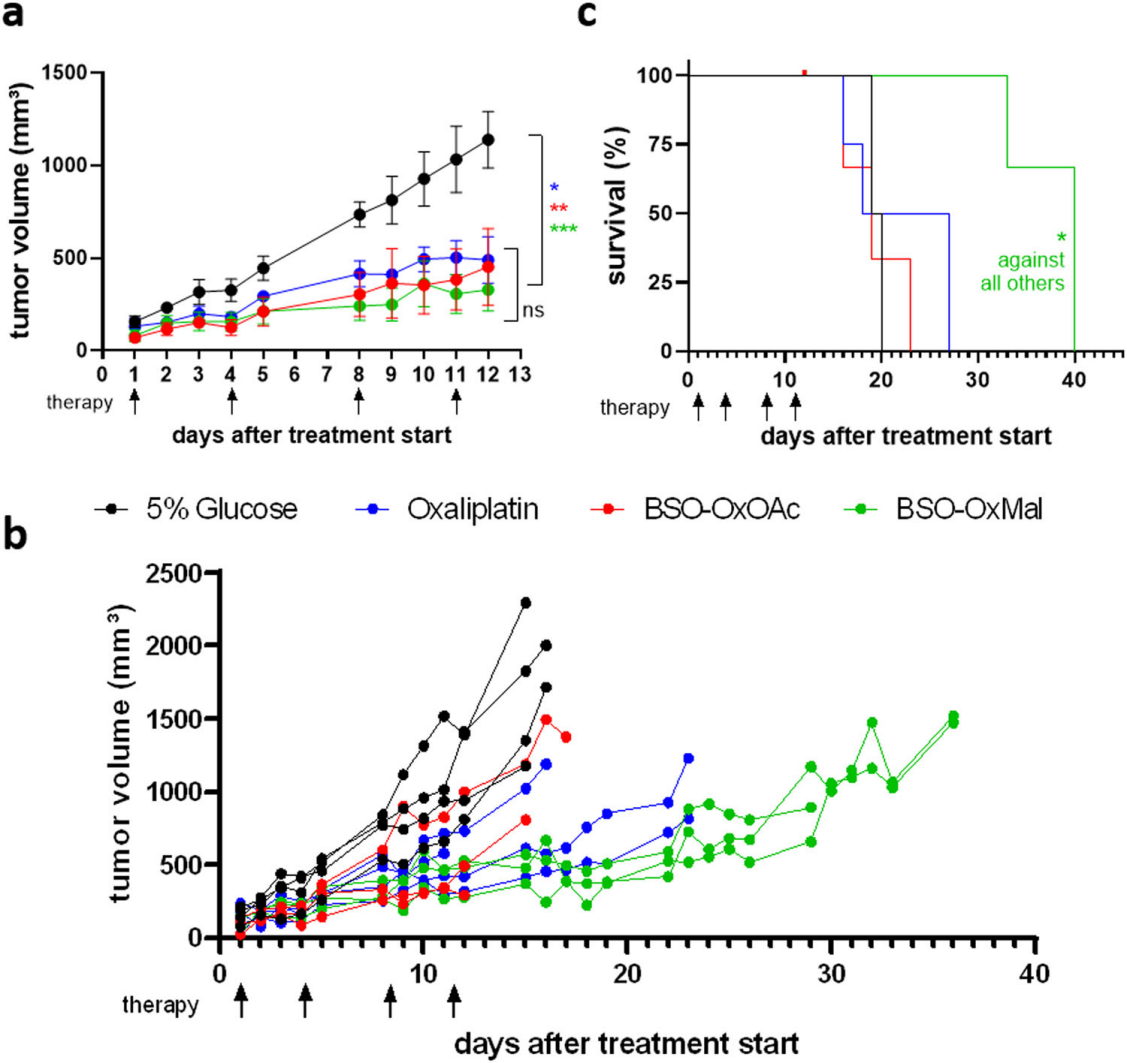
In vivo anticancer activity of **BSO-OxMal** and **BSO-OxOAc** compared to oxaliplatin. Mice bearing CT26 allografts (*n* = 4 per group) were treated twice a week for two weeks (black arrows) with equimolar concentrations of **BSO-OxMal** (23.5 mg/kg), **BSO-OxOAc** (19.1 mg/kg) or oxaliplatin (9 mg/kg). Mean ± SEM (standard error of mean) (**a**) and individual (**b**) tumor volumes were assessed at the indicated time points by caliper measurements (maximal tumor growth inhibition of 61.2%, 51.3% and 51.3% at day 11, for **BSO-OxMal**, **BSO-OxOAc** and oxaliplatin respectively; tumor doubling time extended by 1.8-fold, 1.2-fold, and 1.3-fold, respectively). (**c**) Overall survival of mice based on FELASA guidelines was analyzed by Kaplan Meier curves. Statistical significance was tested using one-way ANOVA (**a**) or Mantel-Cox test (**c**). In all cases: *p < 0.05; **p < 0.01; ***p < 0.001.

As a next step, the antitumor activity of our platinum(IV) prodrugs **BSO-OxMal** and **BSO-OxOAc** was evaluated and compared at equimolar conditions to free oxaliplatin at the maximum tolerated dose (MTD) of 9 mg/kg. Treatment for two weeks (four doses) of the test compounds was well tolerated and no substantial loss of animal body weights was observed (Supplementary Fig. 12). Application of **BSO-OxMal**, **BSO-OxOAc** or oxaliplatin significantly reduced tumor volumes as compared to solvent controls during the first two weeks of treatment without significant differences between the three metal drugs (Fig. 8a). After this period, the first solvent- and **BSO-OxOAc**-treated mice needed to be sacrificed based on tumor size or ulceration, while tumors in the **BSO-OxMal** group stabilized (individual animals shown in Fig. 8b). Consequently, only **BSO-OxMal** treatment significantly increased overall survival (Fig. 8c).

**Fig. 9:**
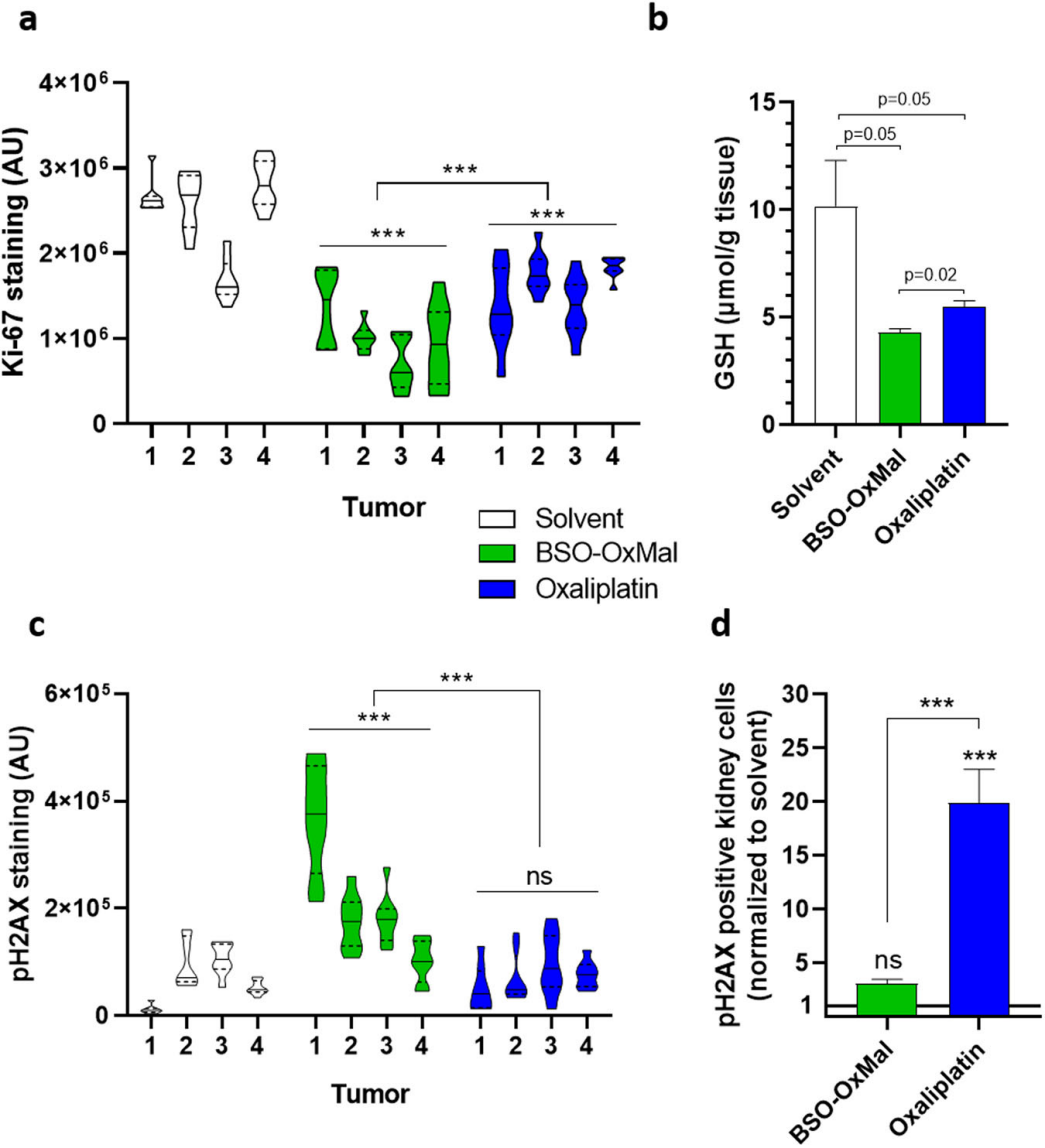
Effects of **BSO-OxMal** and oxaliplatin treatment. Cancer proliferative activity (**a**) and GSH content (**b**) as well as DNA damage in tumor (**c**) and kidney (**d**) tissues. Mice bearing CT26 allografts (*n* = 4 per group) were treated twice a week with equimolar concentrations of **BSO-OxMal** (23.5 mg/kg) or oxaliplatin (9 mg/kg) and sacrificed 24 h after the last drug dosing. Immunohistochemical staining of Ki-67 (**a**) and the DNA damage parameter pH2AX (**c**) were quantified in 8–10 regions of interest (ROI) per tumor within random non-necrotic, viable cancer regions by ImageJ software. In (**d**) cells stained positively for pH2AX in the kidney were counted within 5 ROI per kidney section. Representative images are shown for tumor sections in Supplementary Fig. 14 and for organ sections in Supplementary Fig. 15. In (**b**) tumor GSH content of the respective cancer samples was quantified by a calorimetric method as described in the methods section. Data in (**b**, **d**) are depicted as mean ± SEM (standard error of mean). Statistical significance was tested using one-way ANOVA (**a**, **c**) or Student´s t test (**b**, **d**). In all cases: *p < 0.05; **p < 0.01; ***p < 0.001.

To investigate the factors underlying this promising anticancer activity and the contribution of the released BSO, proliferation and DNA damage markers as well as GSH content in cancer tissues were determined after one week of therapy (Fig. 9, Supplementary Fig. 14). The number of mitotic tumor cells (determined by microscopic counting in hematoxylin-eosin (H&E)-stained sections) was significantly reduced in both oxaliplatin- or **BSO-OxMal**-treated animals as compared to the solvent controls, with an insignificant trend towards the enhanced activity of the platinum(IV) prodrug (Supplementary Fig. 13). However, when quantifying immunohistochemical staining of the proliferation marker Ki-67 in multiple non-necrotic tumor regions, a significantly stronger antiproliferative effect of **BSO-OxMal** as compared to oxaliplatin was detected (Fig. 9a). In addition, both platinum compounds distinctly reduced the content of GSH in the malignant tissue with a significantly stronger effect of **BSO-OxMal** as compared to oxaliplatin (Fig. 9b). Still, the strong influence  of oxaliplatin was unexpected, considering the lack of impact in vitro (compare Supplementary Fig. 10). Nevertheless, the effects of oxaliplatin on cellular GSH metabolism have been suggested previously^45,47^. Additionally, it needs to be considered that in the in vivo setting recurrent oxaliplatin dosing was used. Moreover, GSH levels in cancer tissues might not reflect cancer cells only, but also the tumor microenvironment. Due to the anticancer effect, blood supply and consequently building block delivery for GSH synthesis might be reduced, while in the in vitro situation these compounds are directly delivered in the growth medium. Additionally, not only **BSO-OxMal** but also oxaliplatin distinctly reduced the tumor volume already after one week of treatment (compare Fig. 8). Hence, the reduction of the GSH level by both drugs might also reflect to some extent beginning tumor necrosis. The reduced protection of tumor cells against oxaliplatin-mediated DNA damage by GSH was additionally verified on a functional basis by immunohistochemical staining of the DNA damage marker pH2AX. **BSO-OxMal** treatment-induced distinctly enhanced DNA damage as compared to oxaliplatin also in non-necrotic parts of the tumor (Fig. 9c, Supplementary Fig. 14). Notably, this was in sharp contrast to DNA damage in organ sections of the treated animals. While in the liver no pH2AX staining was observed, oxaliplatin, but not the platinum(IV) prodrug **BSO-OxMal**, induced pH2AX-positive cells in both lung and kidney samples (Supplementary Fig. 15). When counting the number of DNA-damaged cells in multiple kidney sections, this difference was significant (Fig. 9d). These findings are in line with the limited accumulation of **BSO-OxMal** in non-tumor tissues (compare Fig. 7). Despite the fact that the dose-limiting adverse effect of oxaliplatin is mainly peripheral neurotoxicity and not, like in case of cisplatin, nephrotoxicity, also for oxaliplatin oxidative damage in the kidney has been described^48^. Hence, **BSO-OxMal** avoids renal DNA damage despite the suppression of GSH synthesis by BSO, impressively confirming the excellent tumor-targeting of this drug. This is remarkable, considering that GSH has even been suggested to protect healthy tissues from platinum drug-induced DNA damage^21^.

## Conclusion

Successful application of anticancer metal drugs comprising a platinum(II) core, such as cisplatin, oxaliplatin and carboplatin, is limited due to severe side effects and the occurrence of acquired resistance phenotypes^8^. Platinum(IV) prodrug complexes might here represent a promising alternative, but have failed clinical approval so far, at least partly because of unselective activation outside the malignant tissue^10^. Previously, we have shown that albumin-targeting of platinum(IV) complexes via an axial maleimide moiety enhances the plasma half-life and selectivity towards cancerous tissues^14^. Nevertheless, resistance mechanisms such as increased intracellular GSH conjugation would also affect these compounds. Consequently, we herein synthesized oxaliplatin-based triple-action platinum(IV) complexes, containing BSO as an additional ligand in the second axial position besides the albumin-targeting maleimide moiety. This additional bioactive ligand, released at the site of reduction, should specifically inhibit GSH synthesis via GCL in cancer cells, but probably also in cells of the microenvironment, thereby reducing export or degradation of free oxaliplatin. Additionally, the prodrug strategy should improve the pharmacokinetic properties of BSO, characterized as a free drug by short plasma half-life (<2 h) due to fast metabolism and excretion^39^. Hence, we hypothesized, that attaching BSO to a platinum(IV) prodrug enables prolonged plasma circulation and may guide tumor-specific delivery when combined with albumin binding. In order to prove these hypotheses, two platinum(IV) prodrugs, namely albumin-targeting maleimide-bearing **BSO-OxMal**, as well as **BSO-OxOAc**, possessing a biologically inactive acetato ligand, were synthesized. The compound class showed high stability in aqueous solution and inertness towards reductants, making early reduction in the bloodstream or red blood cells rather unlikely. Indeed, **BSO-OxOAc** exerted markedly lowered cytotoxic activity in vitro and required longer exposure time and presence of higher amounts of reducing agents to be activated, thus proving the high stability against premature activation. Nevertheless, already the in vitro accumulation and cytotoxicity data clearly suggested distinct insensitivity of the prodrug against efflux-mediated oxaliplatin resistance. The validity of the prodrug strategy was convincingly proven in CT26 tumor-bearing mice. Indeed, **BSO-OxMal** revealed massively prolonged serum half-life and enhanced tumor selectivity as compared to free oxaliplatin, determined at the level of platinum accumulation by ICP-MS. Accordingly, **BSO-OxMal**- but not **BSO-OxOAc**-treated mice demonstrated significantly prolonged overall survival in comparison to oxaliplatin-treated animals, confirming the importance of specific tumor-targeting of platinum(IV) prodrugs, e.g. via albumin binding, to be functional. Intratumorally, **BSO-OxMal** had a stronger GSH-depleting effect than oxaliplatin and, well in accordance with the role of GSH in drug inactivation, led to significantly increased DNA damage and proliferation arrest. Notably, the platinum(IV) prodrug did not increase DNA damage in lungs and kidneys as observed in case of oxaliplatin treatment, proving that the sensitizing effect of BSO against oxaliplatin-mediated DNA damage is limited specifically to the malignant tissue in case of **BSO-OxMal**.

Summarizing, oxaliplatin therapy implies several clinical problems, like insufficient tumor targeting and, hence, strong adverse effects, limited plasma half-life, and metabolism-based resistance development. Our here presented strategy to combine an oxaliplatin(IV) prodrug, a respective resistance modulator (BSO) as ligand, and maleimide-mediated binding to a natural, endogenous nanocarrier (albumin) represents a promising approach to overcome these limitations.

## Methods

### Synthesis

#### General materials and methods

**Fig. 10:**
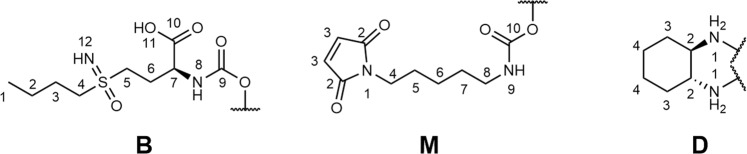
NMR Numbering scheme of the platinum ligands. The letters stand for B - BSO; M - maleimide and D - 1,2-diaminocyclohexane, respectively.

Potassium tetrachloridoplatinate (K_2_PtCl_4_) was purchased from Johnson Matthey (Switzerland) and BSO was acquired from Toronto Research Chemicals (Canada). Water for synthesis was taken from a reverse osmosis system and distilled twice before use. For HPLC measurements Milli-Q water (18.2 MΩ ∙ cm, Merck Milli-Q Advantage, Darmstadt, Germany) was used. Chemicals and solvents were purchased from commercial suppliers (Sigma Aldrich, Merck, Acros, Fluka and Fisher Scientific). Oxaliplatin and the respective platinum(IV) complexes **Ox(OH)**_**2**_ and **Ox(OH)(OAc),** as well as the maleimide ligand **5** were synthesized similarly to methods described in literature^44,49–51^. Electrospray ionization (ESI) mass spectra were recorded on a Bruker amaZon SL ion trap or a Bruker maXis UHR-TOF mass spectrometer in positive and/or negative mode by direct infusion at the Mass Spectrometry Centre of the University of Vienna. One- and two-dimensional ^1^H-, and ^13^C-NMR spectra were recorded on a Bruker AV Neo 500 or AV III 600 spectrometer at 298 K. For ^1^H- and ^13^C-NMR spectra the solvent residual peak was taken as the internal reference. The ^1^H- and ^13^C-NMR spectra of the final compounds are depicted in Supplementary Figs. 1 and 2. NMR numbering of the ligands of the final compounds can be found in Fig. 10. Purification by preparative reverse-phase HPLC was performed on an Agilent 1200 series system using a Waters XBridge C18 column (19 × 250 mm). Elemental analysis measurements were done on a Perkin Elmer 2400 CHN Elemental Analyzer at the Microanalytical Laboratory of the University of Vienna and are within ±0.4%, confirming >95% purity.

#### (2 *S*)-2-((((9*H*-Fluoren-9-yl)methoxy)carbonyl)amino)-4-(butylsulfonimidoyl)butanoic acid (1)

**BSO** (1.5 g, 6.75 mmol) was dissolved in H_2_O (30 mL) and NaHCO_3_ (1.13 g, 13.5 mmol, 2 eq.) was added. Fmoc-Cl (1.66 g, 6.41 mmol, 0.95 eq.) was dissolved in dimethoxyethane (DME, 30 mL) and both solutions were combined and stirred at room temperature (RT) for 20 h. DME was removed under reduced pressure, the remaining residue adjusted to pH 2 with aqueous 1 M HCl and extracted 3-times with ethyl acetate (EtOAc). The combined organic fractions were washed with brine, dried over MgSO_4_ and evaporated to dryness to obtain **1** (2.77 g, 97%) as a white solid.

^1^H NMR (500 MHz, DMSO-*d*_6_) δ 7.90 (d, *J* = 7.5 Hz, 2H), 7.83 (dd, *J* = 8.5, 2.2 Hz, 1H), 7.72 (dd, *J* = 7.3, 2.6 Hz, 2H), 7.43 (t, *J* = 7.5 Hz, 2H), 7.34 (t, *J* = 7.5 Hz, 2H), 4.38–4.28 (m, 2H), 4.24 (t, *J* = 7.0 Hz, 1H), 4.21–4.13 (m, 1H), 3.68–3.48 (m, 4H), 2.34–2.23 (m, 1H), 2.16–2.04 (m, 1H), 1.81–1.66 (m, 2H), 1.47–1.38 (m, 2H), 0.91 (t, *J* = 7.4 Hz, 3H). MS (m/z): calcd. C_23_H_29_N_2_O_5_S (M + H)^+^, 445.18; found, 445.18.

#### *tert*-Butyl (2 *S*)-2-((((9*H*-fluoren-9-yl)methoxy)carbonyl)amino)-4-(butylsulfonimidoyl)butanoate (2)

**1** (1 g, 2.25 mmol) was suspended in *tert*-butyl acetate (30 mL). Perchloric acid (60%) (0.37 mL, 3.37 mmol, 1.5 eq.) was added slowly and the reaction mixture stirred at RT for 2 h. The phases were separated and the aqueous layer extracted twice with EtOAc. The combined organic fractions were washed with brine, dried over MgSO_4_, evaporated to dryness and the crude product purified by flash column chromatography (30% hexane in EtOAc) to obtain **2** (854 mg, 75%) as a white solid.

^1^H NMR (500 MHz, DMSO-*d*_6_) δ 7.90 (d, *J* = 7.6 Hz, 2H), 7.82 (d, *J* = 8.1 Hz, 1H), 7.71 (d, *J* = 6.9 Hz, 2H), 7.42 (t, *J* = 7.4 Hz, 2H), 7.33 (t, *J* = 7.4 Hz, 2H), 4.41–4.25 (m, 2H), 4.23 (t, *J* = 6.9 Hz, 1H), 4.11–4.05 (m, 1H), 3.27–3.15 (m, 4H), 2.23–2.12 (m, 1H), 2.07–1.98 (m, 1H), 1.75–1.61 (m, 2H), 1.45–1.34 (m, 11H) 0.90 (t, *J* = 7.3 Hz, 3H). MS (m/z): calcd. C_27_H_37_N_2_O_5_S (M + H)^+^, 501.24; found, 501.24.

#### *tert*-Butyl (2 *S*)-2-amino-4-(butylsulfonimidoyl)butanoate (3)

**2** (840 mg, 1.68 mmol) was dissolved in 20% piperidine in dimethylformamide (DMF) (v:v) (20 mL) and stirred at RT for 24 h. The solvents were removed *in vacuo* and the crude product purified by flash column chromatography (1% triethylamine (TEA), 10% MeOH in EtOAc) to obtain **3** (446 mg, 95%) as an orange oil.

^1^H NMR (500 MHz, DMSO-*d*_6_) δ 3.47 (b s, 1H), 3.10–2.92 (m, 5H), 2.08–1.97 (m, 1H), 1.90–1.79 (m, 1H), 1.70–1.58 (m, 2H), 1.50–1.34 (m, 11H), 1.14 (t, *J* = 7.2 Hz, 2H), 0.90 (t, *J* = 7.4 Hz, 3H). MS (m/z): calcd. C_12_H_27_N_2_O_3_S (M + H)^+^, 279.17; found, 279.15.

#### *(OC*-6-34)-Acetato(((2 *S*)-1-(*tert*-butoxy)-4-(butylsulfonimidoyl)-1-oxobutan-2-yl)carbamato((1 *R*,2 *R*)-1,2-cyclohexanediamino)oxalatoplatinum(IV) (4a)

To a suspension of **Ox(OH)(OAc)** (170 mg, 0.36 mmol) in anhy. DMF (4 mL) was added *N,N’*-disuccinimidyl carbonate (150 mg, 0.54 mmol, 1.5 eq.) and the reaction mixture was stirred at RT under Ar for 3 h. **3** (120 mg, 0.47 mmol, 1.3 eq.) was added and stirred at 40 °C under Ar for 24 h. The solvent was removed *in vacuo* and the crude product purified by RP-HPLC (21% MeCN (+0.1% HCOOH) in H_2_O ( + 0.1% HCOOH) isocratic; flow rate 25 mL/min) to obtain **4a** (80 mg, 28%) as a white solid.

^1^H NMR (600 MHz, DMSO-*d*_6_) δ 9.49–9.01 (m, 1H), 8.64–8.45 (m, 1H), 8.45–8.15 (m, 2H), 7.21 (dd, *J* = 7.7, 3.6 Hz, 1H), 3.99–3.88 (m, 1H), 3.08–2.79 (m, 4H), 2.61–2.54 (m, 2H), 2.18–2.03 (m, 3H), 2.01–1.89 (m, 4H), 1.71–1.58 (m, 2H), 1.55–1.48 (m, 2H), 1.48–1.31 (m, 13H), 1.23–1.05 (m, 2H), 0.89 (t, *J* = 7.4 Hz, 3H). MS (m/z): calcd. C_23_H_42_N_4_O_11_PtSNa (M + Na)^+^, 800.20; found, 800.21.

#### (*OC*-6-34)-Acetato(((1 *S*)-3-(butylsulfonimidoyl)-1-carboxypropyl)carbamato)((1 *R*,2 *R*)-1,2-cyclohexanediamino)oxalatoplatinum(IV) (BSO-OxOAc)

**4a** (70 mg, 0.09 mmol) was dissolved in dichloromethane (1.8 mL). Trifluoroacetic acid (TFA, 200 µL) was added and the mixture stirred at RT for 6 h. The solvent was removed *in vacuo* and the crude product purified by RP-HPLC (9% MeCN in H_2_O ( + 0.1% HCOOH) isocratic; flow rate 25 mL/min) to obtain **BSO-OxOAc** (40 mg, 58%) as a white solid.

^1^H NMR (600 MHz, DMSO-*d*_6_) δ 9.25 (b s, 1H, D-1), 8.63–8.14 (m, 3H, 3x D-1), 7.15 (dd, *J* = 7.9, 3.4 Hz, 1H, B-8), 4.12–3.98 (m, 1H, B-7), 3.08–2.86 (m, 4H, B-4, B-5), 2.60–2.53 (m, 2H, D-2), 2.18–2.04 (m, 3H, 2H D-3, 1H B-6), 2.03–1.92 (m, 4H, 1H B-6, 3H PtOC(=O)CH_3_), 1.71–1.57 (m, 2H, B-3)), 1.50 (d, *J* = 8.7 Hz, 2H, D-4), 1.46–1.31 (m, 4H, 2H B-2, 2H D-3), 1.22–1.09 (m, 2H, D-4), 0.89 (t, *J* = 7.4 Hz, 3H, B-1). ^13^C NMR (151 MHz, DMSO-d_6_) δ 178.3 (PtO*C*(=O)CH_3_), 173.2 (B-10), 163.9, 163.8 (d, B-9), 163.4, 163.4 (PtO*C*(=O)_2_), 61.1, 60.8 (D-2), 53.4, 53.2 (d, B-4), 52.9, 52.9 (d, B-7), 50.7, 50.6 (d, B-5), 31.0, 30.9 (D-3), 24.7, 24.6 (d, B-6), 24.1, 24.0 (d, B-3), 23.5, 23.5 (D-4), 22.9 (PtOC(=O)*C*H_3_), 21.1 (B-2), 13.6 (B-1). HRMS (m/z): calcd. C_19_H_35_N_4_O_11_PtS (M + H)^+^, 722.1665; found, 722.1647. EA: calcd. C_19_H_34_N_4_O_11_PtS *TFA *H_2_O, C: 29.55, H: 4.37, N: 6.56, S: 3.76; found, 29.27, H: 4.34, N: 6.55, S: 3.81.

#### (*OC*-6-34)-((2 *S*)-1-(*tert*-Butoxy)-4-(butylsulfonimidoyl)-1-oxobutan-2-yl)carbamato(1 *R*,2 *R*)-1,2-cyclohexanediaminohydroxidooxalatoplatinum(IV) (4b)

**Ox(OH)**_**2**_ was suspended in anhy. dimethyl sulfoxide (DMSO, 3 mL) and N,N’-disuccinimidyl carbonate (154 mg, 0.60 mmol, 1.3 eq.) was added as a solution in anhy. DMSO (0.5 mL) over a period of 18 h at RT under Ar. After stirring for 2 h **3** (194 mg, 0.70 mmol, 1.5 eq.) was added and the reaction mixture stirred for 18 h at 40 °C under Ar. The solvent was removed *in vacuo* at 50 °C and the crude product purified by RP-HPLC (21% MeCN (+0.1% HCOOH) in H_2_O ( + 0.1% HCOOH) isocratic; flow rate 25 mL/min) to obtain **4a** (77 mg, 22%) as a white solid.

NMR (500 MHz, DMSO-*d*_6_) δ 9.57–9.03 (m, 1H), 8.40–8.00 (m, 1H), 7.82–7.45 (m, 1H), 7.21–6.93 (m, 1H), 6.90–6.23 (m, 1H), 4.07–3.84 (m, 1H), 3.68–3.57 (m, 1H), 3.05–2.70 (m, 4H), 2.19–1.80 (m, 4H), 1.70–1.57 (m, 2H), 1.57–1.17 (m, 15H), 1.17–0.96 (m, 2H), 0.89 (t, J = 7.1 Hz, 3H); MS (m/z): calcd. C_21_H_40_N_4_O_10_PtSNa (M + Na)^+^, 758.20; found, 758.26.

#### (*OC*-6-24)-((2 *S*)-1-(*tert*-butoxy)-4-(butylsulfonimidoyl)-1-oxobutan-2-yl)carbamato(1 *R*,2 *R*)-1,2-cyclohexanediamino(5-(2,5-dioxo-2,5-dihydro-1*H*-pyrrol-1-yl)pentyl)carbamatooxalatoplatinum(IV) (6)

To a solution of **4b** (78 mg, 0.11 mmol) in anhy. DMF (4 mL) under Ar was added **5** (24 mg, 0.12 mmol, 1.1 eq.) in DMF (1 mL) over 17 h and the reaction mixture was further stirred at RT for 3 h. The solvent was removed *in vacuo* and the crude product purified by RP-HPLC (31% MeCN (+0.1% HCOOH) in H_2_O ( + 0.1% HCOOH) isocratic; flow rate 25 mL/min) to obtain **6** (48 mg, 48%) as a white solid.

^1^H NMR (600 MHz, DMSO-*d*_6_) δ 9.97–9.49 (m, 1H), 9.49–8.88 (m, 1H), 8.71–8.23 (m, 2H), 7.28–7.16 (m, 1H), 6.99 (s, 2H), 6.82–6.29 (m, 1H), 4.15–3.86 (m, 1H), 3.79–3.56 (m, 1H), 3.40–3.36 (m, 2H), 3.07–2.75 (m, 6H), 2.60–2.54 (m, 2H), 2.21–2.10 (m, 2H), 2.09–1.99 (m, 1H), 1.98–1.89 (m, 1H), 1.71–1.56 (m, 2H), 1.55–1.49 (m, 2H), 1.48–1.42 (m, 2H), 1.42–1.29 (m, 15H), 1.23–1.03 (m, 4H), 0.89 (t, *J* = 7.4 Hz, 3H). MS (m/z): calcd. C_31_H_53_N_6_O_13_PtS (M + H)^+^, 944.30; found, 944.31.

#### (*OC*-6-24)-((1 *S*)-3-(Butylsulfonimidoyl)-1-carboxypropyl)carbamato(1 *R*,2 *R*)-1,2-cyclohexanediamino(5-(2,5-dioxo-2,5-dihydro-1*H*-pyrrol-1-yl)pentyl)carbamatooxalatoplatinum(IV) (BSO-OxMal)

**6** (46 mg, 0.05 mmol) was dissolved in dichloromethane (1.6 mL). TFA (400 µL) was added and the mixture stirred at RT for 5 h. The solvent was removed *in vacuo* and the crude product purified by RP-HPLC (23% MeCN (+0.1% TFA) in H_2_O ( + 0.1% TFA) isocratic; flow rate 25 mL/min) to obtain **BSO-OxMal** (41 mg, 82%) as a white solid.

^1^H NMR (600 MHz, DMSO-*d*_6_) δ 9.90–9.36 (m, 1H, D-1), 9.36–8.97 (m, 1H, D-1), 8.72–8.46 (m, 1H, D-1), 8.46–8.22 (m, 1H, D-1), 7.25–7.12 (m, 1H, B-8), 7.00 (s, 2H, M-3), 6.85–6.74 (m, 1H, M-9), 4.15–3.98 (m, 1H, B-7), 3.58–3.43 (m, 6H, B-4, B-5, M-4), 2.95–2.75 (m, 2H, M-8), 2.60–2.54 (m, 2H, D-2), 2.27–2.11 (m, 3H, 2H D-3, 1H B-6), 2.11–1.93 (m, 1H, B-6), 1.79–1.64 (m, 2H, B-3), 1.55–1.49 (m, 2H, D-4), 1.49–1.30 (m, 8H, 2H M-5, 2H M-7, 2H B-2, 2H D-3), 1.21–1.06 (m, 4H, 2H D-4, 2H M-6), 0.91 (t, *J* = 7.4 Hz, 3H, B-1). ^13^C NMR (151 MHz, DMSO-*d*_6_) δ 172.8 (B-10), 171.1 (M-2), 164.3 (M-10), 163.9 (B-9), 163.4, 163.3 (PtO*C*(=O)_2_), 157.9, 157.7 (TFA), 134.5 (M-3), 61.1, 61.0 (D-2), 52.6 (B-7), 51.9, 51.7 (d, B-4), 49.6 (B-5), 40.7 (M-8), 37.0 (M-4), 31.0, 31.0 (D-3), 29.0 (M-7), 27.7 (M-5), 23.9, 23.7 (d, B-6), 23.6, 23.5 (D-4), 23.4 (M-6), 23.3 (B-3), 20.8 (B-2), 13.4, 13.4 (d, B-1). HRMS (m/z): calcd. C_27_H_44_N_6_O_13_PtS (M + H)^+^, 888.2408; found, 888.2410. EA: calcd. C_27_H_44_N_6_O_13_PtS* TFA *H_2_O, C: 34.15, H: 4.65, N: 8.24, S: 3.14; found, 33.96, H: 4.48, N: 8.49, S: 2.85.

#### Stability UHPLC measurements

For stability measurements **BSO-OxOAc** (1 mM) was dissolved in phosphate buffer (100 mM, pH 7.4). For reduction experiments **BSO-OxOAc** (2 mM) was dissolved in phosphate buffer (500 mM, pH 7.4). A 20 mM solution of AA in the same buffer was added in a 1:1 ratio. The samples were immediately measured on a Dionex UltiMate 3000 RS UHPLC system equipped with a Waters Acquity UPLC® BEH C18 column (3×50 mm, pore size 1.7 µm), and absorption detection at 230 nm with incubation at 20 °C (Gradients: Method A: 0.0−0.5 min: 5% eluent B in eluent A; 0.5–5.5 min: 5-95% eluent B; 5.5–8.0 min: 95 % eluent B (eluent A = 0.1% formic acid in water, eluent B = 0.1 % formic acid in acetonitrile), method B: 0.0 − 0.5 min: 5% eluent B in eluent A; 0.5–5.5 min: 5-95% eluent B; 5.5–7.1 min: 95 % eluent B (eluent A = 0.1% formic acid in water, eluent B = 0.1 % formic acid in methanol). Data points were taken every 30 min for 6 h and at 24 h and 24.5 h.

#### SEC-ICP-MS measurements

Fetal calf serum was purchased from Sigma-Aldrich and buffered with 150 mM phosphate buffer (pH 7.4) to guarantee a stable pH. The platinum(IV) complexes were dissolved in 150 mM phosphate buffer (pH 7.4) to 1 mM and diluted 1:10 in serum to obtain a final concentration of 100 μM. The samples were then incubated in the autosampler at 37 °C for 24 h and analyzed at 0 h, 1 h, 4 h, and 24 h. Between each sample, a pure water blank was measured. For SEC-ICP-MS measurements, an Agilent 1260 Infinity system coupled to an Agilent 7800 ICP-MS equipped with a dynamic reaction cell was used. Oxygen (purity 5.5, Messer AustriaGmbH, Gumpoldskirchen, Austria) was used as reaction gas at 30% and 0.9 L/min flow. HPLC parameters are given in Table 1, and ICP-MS operation parameters are given in Table 2.

**Table 1:** SEC-HPLC parameters for SEC-ICP-MS measurements.

Instrument	Agilent 1260 Infinity
HPLC column	Acquity UPLC BEH 200 Å 1.7 µm, 4.6×150 mm
Eluent	50 mM CH_3_COONH_4_, pH = 6.8
Flow rate	400 µL/min
Injection volume	5 µl
Column temperature	37 °C
Autosampler temperature	37 °C

**Table 2:** ICP-MS measurement parameters.

Instrument	ICP-MS Agilent 7800
RF power (W)	1550
Cone material	Nickel
Carrier gas (L/min)	1.08
Plasma gas (L/min)	15
Monitored isotopes	^185^Re, ^195^Pt, ^196^Pt
Integration time [s]	0.1
Number of sweeps	100
Number of replicates	10

#### Cell lines, cell culture reagents, and drug treatments

All reagents were purchased from Sigma-Aldrich (St. Louis, MO, USA) unless specified otherwise. The human and murine colorectal carcinoma cell lines HCT116 (Expasy accession: CVCL_0291) and CT26 (CVCL_7254), respectively, were obtained from the American Type Culture Collection (ATCC) (Rockville, MD, USA), and the human ovarian cancer cell line A2780 (CVCL_0134, product number 93112519) together with its cisplatin-resistant subline A2780/Cis (93112517) from Sigma Aldrich. The oxaliplatin-resistant subline of HCT116 cells was generated as previously published^47^. Human colorectal fibroblasts (F331)^52^ were generously donated by Prof. Brigitte Marian, human telomerase-immortalized blood endothelial cells (BEC)^53^ and the immortalized human keratinocyte cell line (HaCat; CVCL 0038)^54^ were derived as published. Cells were cultured in McCoy’s 5 A medium (HCT116, HCT116/OxR), RPMI 1640 (A2780, A2780/Cis, HaCat), in DMEM (F331), or in DMEM/F12 (Dulbecco’s Modified Eagle’s Medium/Ham’s Nutrient Mixture F12) (CT26). BEC cells were grown in EGM-2 endothelial growth medium (Clonetics, San Diego, CA). All media were supplemented with 10% heat-inactivated fetal calf serum (BioWest, Riverside, MO) and cells were kept at 37 °C and 5% CO_2_ in a humidified atmosphere. All cell lines were used at passage numbers 3–10 from re-establishment, were authenticated by STR (Eurofins Genomics) and/or array-CGH and were regularly tested for *Mycoplasma* contamination. All investigated compounds were dissolved in DMSO as 10 mM stock solutions and were stored at –20 °C. Dilutions in culture media supplemented with 10% fetal calf serum were made immediately before the experiments at the indicated concentrations. Corresponding dilutions of DMSO were used as untreated vehicle controls^47^.

#### Cell viability

Cells were seeded (3–4 ×10^3^ cells/well) in 96-well plates in 100 µL and after 24 h recovery treated with 100 µL of compounds at the indicated concentrations in triplicates. After 72 h drug exposure cell viability was determined by 3-(4,5-dimethylthiazol-2-yl)-2,5-diphenyltetrazolium bromide (MTT)-vitality assay (EZ4U, Biomedica, Vienna, Austria) following the manufacturer’s recommendations, as published^55^. Absorbance was measured at 450 nm (at 620 nm as reference) using the multimode plate reader Tecan Infinite 200 Pro (Zurich, Switzerland). Cytotoxicity was expressed as IC_50_ values calculated from full dose-response curves using the 4 parameter nonlinear regression model of GraphPad Prism 8 software (La Jolla, CA, USA).

#### In vitro platinum cell uptake

Cells were seeded at 1.5 ×10^5^ cells/well in six-well plates in 1.5 mL and after 24 h recovery treated with 500 µL of compounds at the indicated concentrations in triplicates. After 3 h drug exposure, cells were detached, washed twice with phosphate-buffered saline (PBS), dried at RT for 15 min and lysed in 100 µL HNO_3_ (69%, Rotipuran Supra, Carl Roth, Karlsruhe, Germany) for 1 h at RT. The lysate was added to fresh tubes containing 200 µL ddH_2_O. ICP-MS measurements were performed with an Agilent 8800 ICP-MS/MS instrument (Agilent Technologies, Tokyo, Japan). The sample introduction system consisted of a MicroMist nebulizer (200 μL min^−1^ nominal sample uptake) and a quartz double-pass spray chamber. The instrument was tuned on a daily basis in order to achieve maximum sensitivity, low oxide formation, and low doubly charged ratios. Platinum was monitored in no gas mode with an integration time of 0.3 s. The flow injection measurements were performed with a bioinert HPLC 1260 (Agilent Technologies, Waldbronn, Germany), which was directly connected to the nebulizer of the ICP-MS/MS instrument with a PEEK tubing. The following chromatographic conditions were used: injection volume: 10 µL; flow rate: 0.30 mL min^-1^; isocratic elution; CH_3_COONH_4_ (50 mM, pH = 6.8) aqueous solution was employed as mobile phase. The data were recorded and evaluated with the Agilent MassHunter Chromatography software package, MassHunter 4.6., 2019.

#### Colony formation

Cells were seeded at low densities of 1 × 10^3^ cells/well in 24-well plates in 500 µL and after 24 h recovery treated with 100 µL of compounds at the indicated concentrations in duplicates. Following drug exposure time of 4 or 10 days, cells were washed with PBS, fixed with ice-cold methanol for 30 min at 4 °C and stained with crystal violet, as published^56^. Digital photographs were taken using a Nikon D3200 camera and processed as well as staining density quantified with ImageJ software (NIH).

#### In vivo allograft experiments

Eight-to nine-week-old female *BALB/c* mice bred in-house (originally Janvier) were kept in pathogen-free conditions and controlled environment with 12 h light-dark cycle. CT26 cells (5 × 10^5^ in 50 µL serum-free RPMI medium) were injected subcutaneously (s.c.) into the right flank of the animals. When all tumors were measurable (at day 5-7), all animals (n = 4 per group) were treated intravenously (i.v.) (100 µL/20 g) with solvent (5% glucose) or compounds (dissolved in 5% glucose) at concentrations equimolar to 9 mg/kg oxaliplatin (**BSO-OxMal**: 23.5 mg/kg, **BSO-OxOAc**: 19.1 mg/kg) twice a week for two weeks, using 30 gauze needles. Tumor growth was evaluated by daily recording of tumor size by caliper measurement. In case of the **BSO-OxOAc** and **BSO-OxMal** subgroups, one animal each had to be removed early from analysis due to a fighting-related injury, and thus was censored in survival analyses. In all cases, animals did not die spontaneously, but were sacrificed by cervical dislocation based on Federation of European Laboratory Animal Science Associations (FELASA) endpoints. The reasons were primarily either enhanced tumor size or ulceration. For drug distribution (digestion and ICP-MS measurement details see below), histological and GSH determination experiments, mice were treated twice during one week and sacrificed 24 h after the last drug dosing. The animal experiments were performed according to the regulations of the Ethics Committee for the Care and Use of Laboratory Animals at the Medical University Vienna (proposal number BMBWF-V/3b 2020-0.380.502).

#### Immunohistochemical staining of tumor and organ sections

Formalin-fixed, paraffin-embedded tissue sections were prepared from tumors and animal organs (animal experiment details see above) and H&E stains performed by standard methods. Mouse-specific Ki-67 rabbit mAb (#12202) and mouse-specific pH2AX rabbit mAb (#9718) were purchased from Cell Signaling Technology (Beverly, MA, USA) and used following the manufacturer’s recommendations in a dilution of 1:200 (Ki-67) and 1:500 (pH2AX) for immunohistochemical staining.

#### In vivo serum pharmacokinetic and organ distribution

Serum pharmacokinetics were determined after application of a single dose of oxaliplatin (9 mg/kg) as compared to **BSO-OxMal** at the equimolar dose (23.5 mg/kg) in tumor-free, male *BALB/c* mice (*n* = 4/experimental group). Blood was drawn after 5 min, 30 min, 6 h, 24 h, 72 h, 1 and 2 weeks from the facial vein as published^57^. Serum was isolated by centrifugation at 900 g for 10 min for two times and stored at –80 °C. In addition, blood samples and tissues from the allograft experiment after the two-weeks therapy experiment were analyzed. Digestion of tissue (approx. 10–30 mg gravimetrically weighted) was performed with 2 mL of approx. 20% nitric acid and 100 µL H_2_O_2_ using an open vessel graphite digestion system (coated graphite heating plate, coated sample holder-top for 25 mL vials, perfluoroalkoxy (PFA) vials and PFA lids; Labter, ODLAB; AHF Analysentechnik AG; Germany). HNO_3_ (67–69%, suprapur for trace metal analysis, NORMATOM; VWR international, Austria) and conc. H_2_O_2_ suprapur (Merck, 30%) were used without further purification. Ultrapure water (18.2 MΩ cm, Milli-Q Advantage, Darmstadt, Germany) was used for all dilutions for ICP-MS measurements. Platinum and rhenium standards for ICP-MS measurements were derived from CPI International (Amsterdam, The Netherlands). The platinum content was determined using an Agilent 7800 ICP-MS instrument. The ICP-MS Agilent 7800® (Agilent Technologies, Tokyo, Japan) was equipped with an Agilent SPS 4 autosampler (Agilent Technologies, Tokyo, Japan) and a MicroMist nebulizer at a sample uptake rate of approx. 0.2 mL/min. The Agilent MassHunter® software package (Workstation Software, version C.01.04, Build 544.17, Patch 3, 2018) was used for data processing. The experimental parameters for ICP-MS are summarized in Table 2. The instrument was tuned on a daily basis to achieve maximum sensitivity. In case of the single-dose experiment, serum area-under the curve (AUC) and serum half-life (one-phase and two-phase decay models) were determined using the respective non-linear fit regression models of GraphPad Prism software.

#### GSH content

GSH levels in HCT116 and HCT116/OxR cells (7.5 × 10^4^ cells per well in 96-well plates, in triplicate), or in CT26 tumor tissues (25–50 mg/sample) were determined using the luminescence-based detection system GSH/GSSG-Glo^TM^ assay (Promega, Madison, WI) following the manufacturer’s recommendations. Quantifications are based on the comparison with a GSH standard curve delivered in the assay and normalized to the total protein content of the samples determined by the Micro BCA^TM^ protein assay kit (Thermo Scientific).

#### Statistical analysis

All data are expressed as mean ± standard deviation (SD), unless stated otherwise. Data of independent experiments performed in triplicates were analyzed using GraphPad Prism 8.0 software (GraphPad Software, Inc.). Dose-response curves were analyzed by four parameter logistic (4PL) nonlinear regression model and corresponding IC_50_ values were derived by interpolation. One- or two-way analysis of variance (ANOVA) with multiple comparison test (Sidak or Tukey) was performed for statistical evaluation if not stated otherwise, *p* values < 0.05 were considered statistically significant [*p* < 0.05(*); < 0.01 (**); < 0.001 (***)].

### Reporting summary

Further information on research design is available in the Nature Research Reporting Summary linked to this article.

## Supplementary information

Reporting Summary

Supplementary Information

## Data Availability

Relevant data are available from the corresponding authors on reasonable request.
