# Supplementary material for: A platinum(IV) prodrug strategy to overcome glutathione-based oxaliplatin resistance
Source: Commun Chem. 2022 Apr 6;5:46. doi: 10.1038/s42004-022-00661-z (PMC9814792; doi:10.1038/s42004-022-00661-z)
Supplement: Supplementary file 1 — Reporting Summary [file 42004_2022_661_MOESM1_ESM.pdf]

nature research

Corresponding author(s): Christian Kowol

Last updated by author(s): Feb 22, 2022

Reporting Summary

Nature Research wishes to improve the reproducibility of the work that we publish. This form provides structure for consistency and transparency in reporting. For further information on Nature Research policies, see our Editorial Policies and the Editorial Policy Checklist.

Statistics

For all statistical analyses, confirm that the following items are present in the figure legend, table legend, main text, or Methods section.

n/a	Confirmed	
☐	☒	The exact sample size (*n*) for each experimental group/condition, given as a discrete number and unit of measurement
☐	☒	A statement on whether measurements were taken from distinct samples or whether the same sample was measured repeatedly
☐	☒	The statistical test(s) used AND whether they are one- or two-sided<br>*Only common tests should be described solely by name; describe more complex techniques in the Methods section.*
☒	☐	A description of all covariates tested
☒	☐	A description of any assumptions or corrections, such as tests of normality and adjustment for multiple comparisons
☒	☐	A full description of the statistical parameters including central tendency (e.g. means) or other basic estimates (e.g. regression coefficient) AND variation (e.g. standard deviation) or associated estimates of uncertainty (e.g. confidence intervals)
☒	☐	For null hypothesis testing, the test statistic (e.g. *F*, *t*, *r*) with confidence intervals, effect sizes, degrees of freedom and *P* value noted<br>*Give P values as exact values whenever suitable.*
☒	☐	For Bayesian analysis, information on the choice of priors and Markov chain Monte Carlo settings
☒	☐	For hierarchical and complex designs, identification of the appropriate level for tests and full reporting of outcomes
☒	☐	Estimates of effect sizes (e.g. Cohen's *d*, Pearson's *r*), indicating how they were calculated

Our web collection on statistics for biologists contains articles on many of the points above.

Software and code

Policy information about availability of computer code

Data collection	One- and two-dimensional 1H-, and 13C-NMR spectra were recorded on a Bruker AV Neo 500 or AV III 600 spectrometer at 298 K. Electrospray ionization (ESI) mass spectra were recorded on a Bruker amaZon SL ion trap or a Bruker maXis UHR-TOF mass spectrometer. Elemental analysis measurements were done on a Perkin Elmer 2400 CHN Elemental Analyzer.
Data analysis	GraphPad Prism (version 8.0.1; GraphPad Software, San Diego, CA); ImageJ 1.51f and 1.52g; Zeiss Zen 2.1 (Carl Zeiss); Zeiss Zen 2010 B SP1; TopSpin 4.0.5 (Bruker Software); OriginPro 2019 (9.6.0.172)

For manuscripts utilizing custom algorithms or software that are central to the research but not yet described in published literature, software must be made available to editors and reviewers. We strongly encourage code deposition in a community repository (e.g. GitHub). See the Nature Research guidelines for submitting code & software for further information.

Data

Policy information about availability of data

All manuscripts must include a data availability statement. This statement should provide the following information, where applicable:

- Accession codes, unique identifiers, or web links for publicly available datasets
- A list of figures that have associated raw data
- A description of any restrictions on data availability

The authors declare that all other data supporting the findings of this study are available within the article and its Supplementary Information files, or are available from the authors upon request.

Field-specific reporting

Please select the one below that is the best fit for your research. If you are not sure, read the appropriate sections before making your selection.

☒ Life sciences ☐ Behavioural & social sciences ☐ Ecological, evolutionary & environmental sciences

For a reference copy of the document with all sections, see nature.com/documents/nr-reporting-summary-flat.pdf

Life sciences study design

All studies must disclose on these points even when the disclosure is negative.

Sample size	All experiments were repeated independently at least twice and were performed normally in three technical replicates in each case to allow for statistical evaluation of data obtained.
Data exclusions	None of the experiments depicted data points were excluded.
Replication	Experiments and results were in all cases widely reproducible at least 3 times.
Randomization	Randomization is not applicable to this study, as all experiments depicted were performed in vitro (cell-free) or in cell culture.
Blinding	not applicable

Reporting for specific materials, systems and methods

We require information from authors about some types of materials, experimental systems and methods used in many studies. Here, indicate whether each material, system or method listed is relevant to your study. If you are not sure if a list item applies to your research, read the appropriate section before selecting a response.

Materials & experimental systems

n/a	Involved in the study	
☐	☒	Antibodies
☐	☒	Eukaryotic cell lines
☒	☐	Palaeontology and archaeology
☐	☒	Animals and other organisms
☒	☐	Human research participants
☒	☐	Clinical data
☒	☐	Dual use research of concern

Methods

n/a	Involved in the study	
☒	☐	ChIP-seq
☒	☐	Flow cytometry
☒	☐	MRI-based neuroimaging

Antibodies

Antibodies used	Mouse specific Ki-67 rabbit mAb (#12202) and mouse specific pH2AX rabbit mAb (#9718) were purchased from Cell Signaling Technology (Beverly, MA, USA) and were used following the manufacturer's recommendations in a dilution of 1:200 (Ki-67) and 1:500 (pH2AX) for immunohistochemical staining.
Validation	Manufacturer information

Eukaryotic cell lines

Policy information about cell lines

Cell line source(s)	The human and murine colorectal carcinoma cell lines HCT116 and CT26, respectively, were obtained from the American Type Culture Collection (ATCC) (Rockville, MD, USA), and the human ovarian cancer cell line A2780 together with its cisplatin-resistant subline A2780/Cis from Sigma Aldrich. Human colorectal fibroblasts (F331) were generously donated by Prof. Brigitte Marian, human telomerase-immortalized blood endothelial cells (BEC) (Paur, J. et al. Hepatology 62, 1767-1778 (2015) and the immortalized human keratinocyte cell line (HaCat; CVCL 0038) were derived as published (Elbling, L. et al. Toxicol. Lett. 205, 173-182 (2011).
Authentication	Authentication was done at the beginning of the study by STR (Eurofins Genomics) and arrayCGH (in our lab).
Mycoplasma contamination	Cells regularly tested on Mycoplasma contamination by PCR (Mycoplasma kit, Sigma Aldrich)
Commonly misidentified lines (See ICLAC register)	No misidentified lines were used.

Animals and other organisms

Policy information about studies involving animals; ARRIVE guidelines recommended for reporting animal research

Laboratory animals	Eight-to nine-week-old female Balb/c mice were bread in-house (originally Harlan) and were kept in pathogen-free conditions and controlled environment with 12 h light-dark cycle.
Wild animals	no
Field-collected samples	no
Ethics oversight	The animal experiments were performed according to the regulations of the Ethics Committee for the Care and Use of Laboratory Animals at the Medical University Vienna (proposal number BMBWF-V/3b 2020-0.380.502).

Note that full information on the approval of the study protocol must also be provided in the manuscript.
