# Supplementary material for: A platinum(IV) prodrug strategy to overcome glutathione-based oxaliplatin resistance
Source: Commun Chem. 2022 Apr 6;5:46. doi: 10.1038/s42004-022-00661-z (PMC9814792; doi:10.1038/s42004-022-00661-z)
Supplement: Supplementary file 2 — Supplementary Information [file 42004_2022_661_MOESM2_ESM.pdf]

A platinum(IV) prodrug strategy to overcome glutathione-based oxaliplatin resistance

Philipp Fronik,[1‡] Michael Gutmann,[2,3‡] Petra Vician,[2] Mirjana Stojanovic,[2] Alexander Kastner,[1] Petra Heffeter,[2,3] Christine Pirker,[2,3] Bernhard K. Keppler,[1,3] Walter Berger,[2,3*] and Christian R. Kowol[1,3*]

[1] University of Vienna, Faculty of Chemistry, Institute of Inorganic Chemistry, Waehringer Strasse 42, 1090 Vienna, Austria

[2] Institute of Cancer Research and Comprehensive Cancer Center, Medical University of Vienna, Borschkegasse 8a, 1090 Vienna, Austria

[3] Research Cluster “Translational Cancer Therapy Research”, 1090 Vienna, Austria

‡ These authors contributed equally to this publication.

* Corresponding authors: E-mail addresses: christian.kowol@univie.ac.at; walter.berger@meduniwien.ac.at

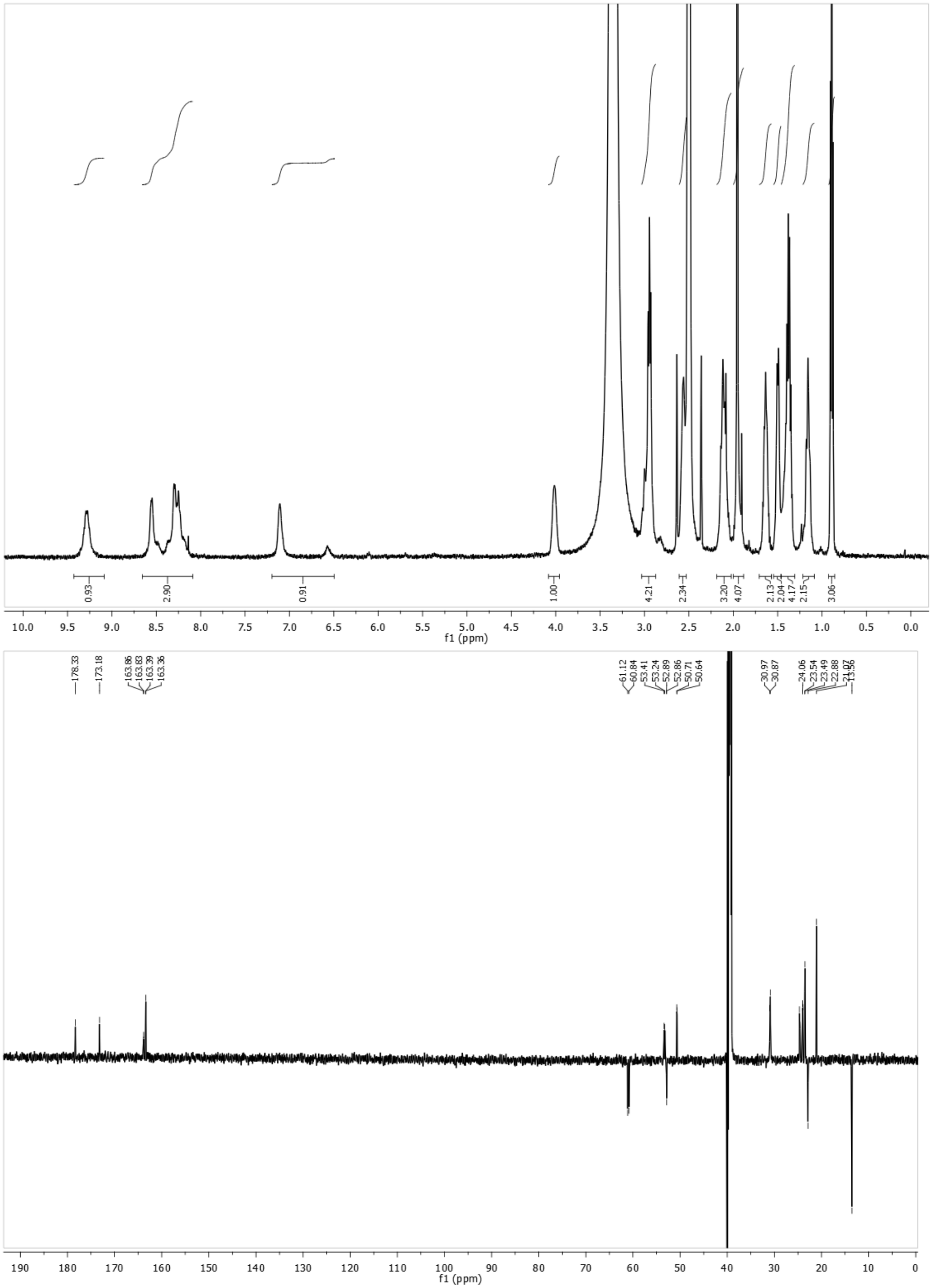

Supplementary Figure 1. ^{1}H and ^{13}C-NMR spectra of **BSO-OxOAc**. The ^{1}H-peak in the range between 6.5 and 7.2 ppm is split due to the different orientations of the carbamate moieties at the platinum core.

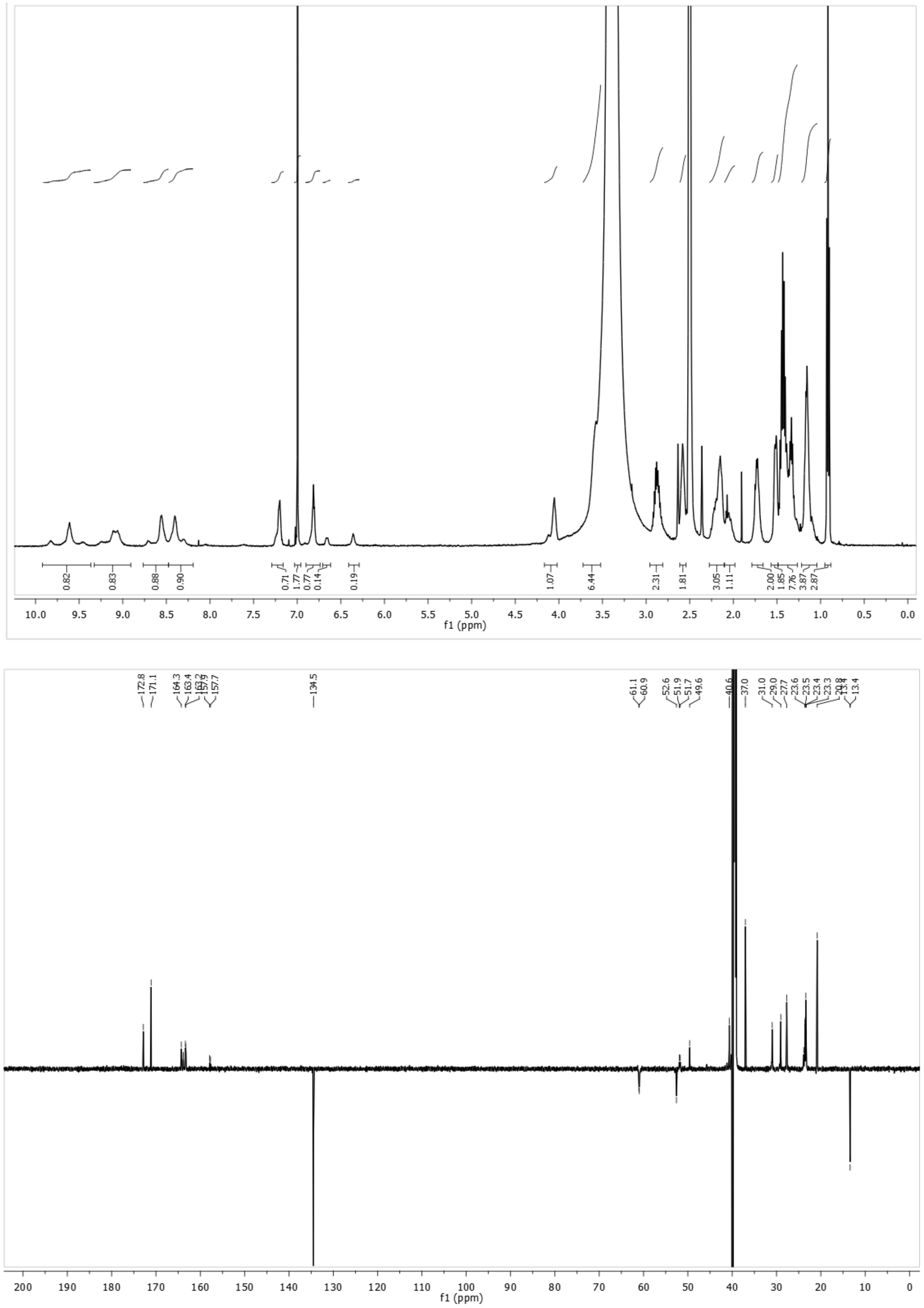

Supplementary Figure 2. ^{1}H and ^{13}C-NMR spectra of **BSO-OxMal**. The ^{1}H-peaks at >8 and in the range between 6.2 and 7.3 ppm are split due to the different orientations of the carbamate moieties at the platinum core.

a

b

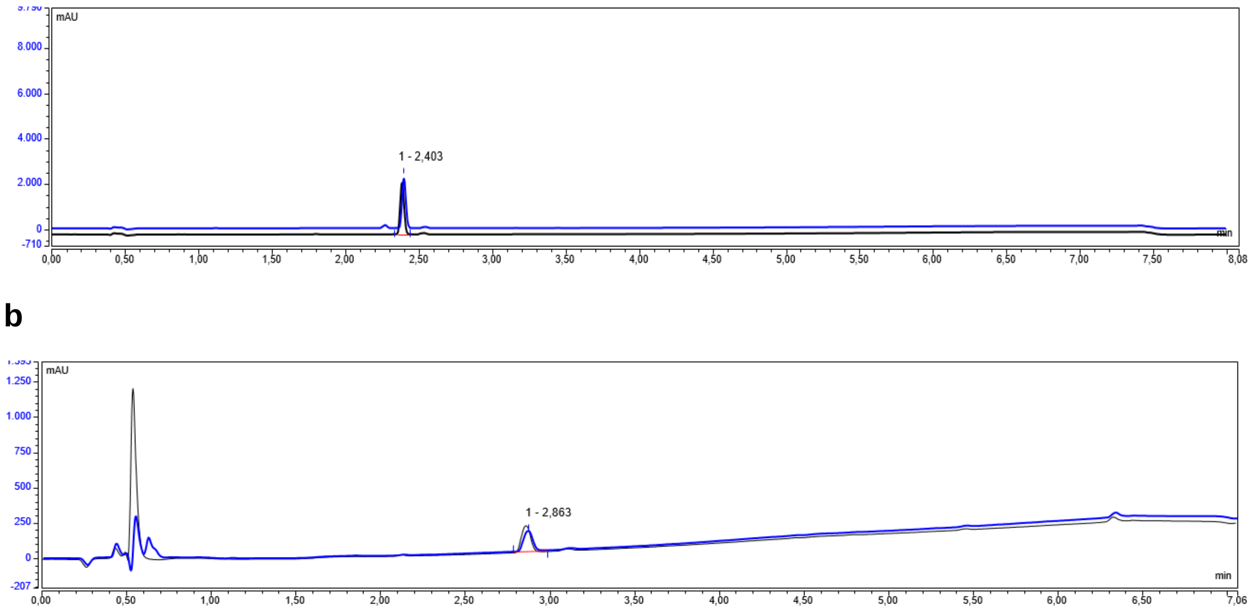

Supplementary Figure 3. (a) UHPLC chromatograms (method A) of the stability of **BSO-OxOAc** (1 mM). Black: t=0 (peak integral = 70.47 mAU), blue: t=24 h (peak integral = 66.25 mAU). (b) UHPLC chromatograms (method B) of the reduction of **BSO-OxOAc** (1 mM) in presence of 10 mM AA. Black: t=0 (peak integral = 11.87 mAU), blue: t=24 h (peak integral = 9.1 mAU).

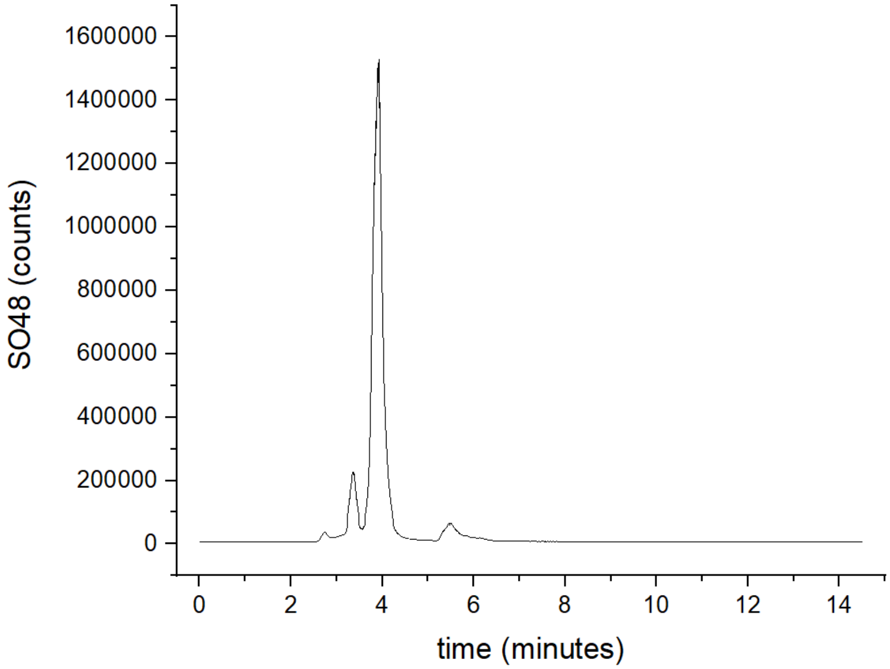

Supplementary Figure 4. Sulfur-ICP-MS trace of fetal calf serum (+150 mM phosphate buffer, pH 7.4). The large peak at t_R = 4 min indicates albumin.

Supplementary Table 1: Quantitative analysis of **BSO-OxOAc** and **BSO-OxMal** binding to the albumin-fraction, obtained by integration of the respective SEC-ICP-MS peaks in the high and low molecular weight fraction of Figure 3.

	**BSO-OxOAc**		**BSO-OxMal**	
t (h)	bound	free	bound	free
0	0%	100%	76%	24%
1	0%	100%	100%	0%
4	0%	100%	100%	0%
24	0%	100%	97%	3%

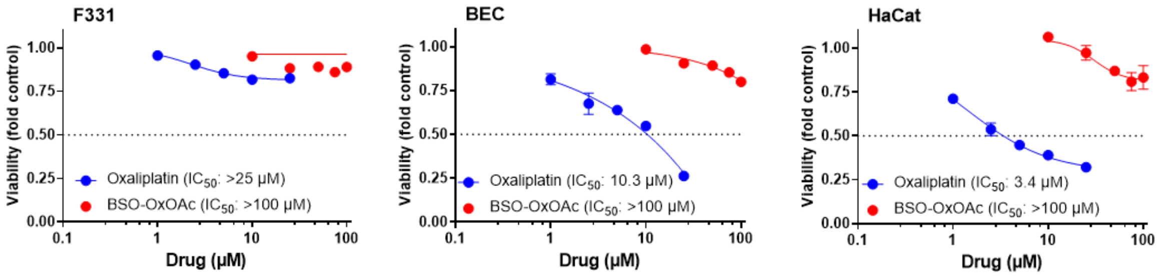

Supplementary Figure 5. Impact of **BSO-OxOAc** as compared to oxaliplatin on the viability of the indicated non-malignant cell lines. Impact of a 72 h continuous drug exposure on the viability of human fibroblast (F331), blood endothelial (BEC) and keratinocyte (HaCat) cells. One representative experiment out of at least three performed in triplicate is shown.

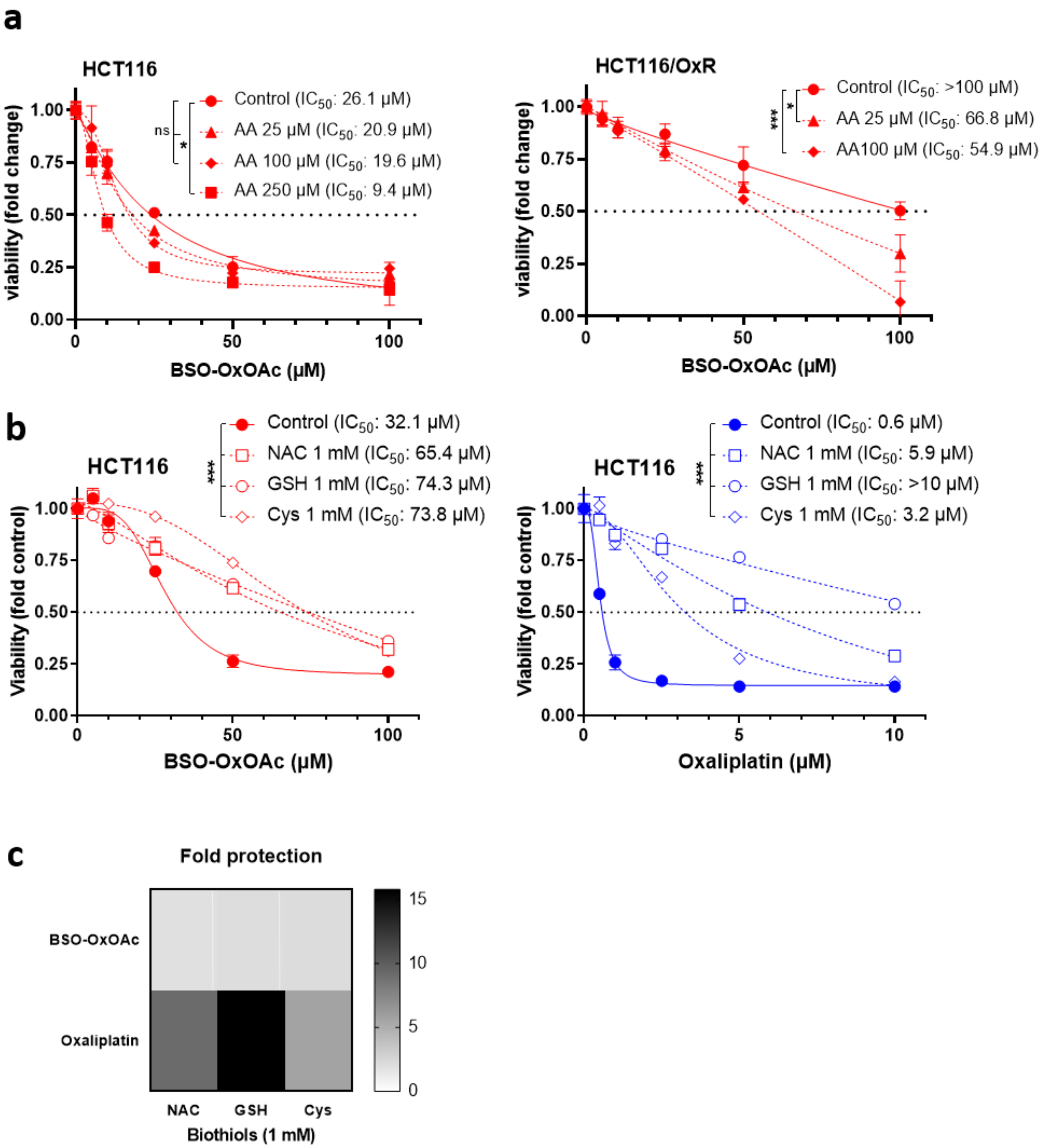

Supplementary Figure 6. Impact of reducing agents on the activity of **BSO-OxOAc** as compared to oxaliplatin on the viability of the human colon cancer cell line HCT116 and an oxaliplatin-resistant subline HCT116/OxR. (a) Impact of a 72 h continuous drug exposure in combination with ascorbic acid (AA) on the viability of HCT116 and HCT116/OxR. (b) Impact of a 72 h continuous drug exposure in combination with NAC, GSH or cysteine (Cys) on the viability of HCT116 cells. One representative experiment out of at least three performed in triplicate is shown. (c) Heat map of the fold protection factors of the experiment shown in (b). IC_{50} values of the combination groups were divided by those of the indicated platinum compounds alone. Statistical

significance was tested using two-way ANOVA. *, p<0.05; ***, p<0.001.

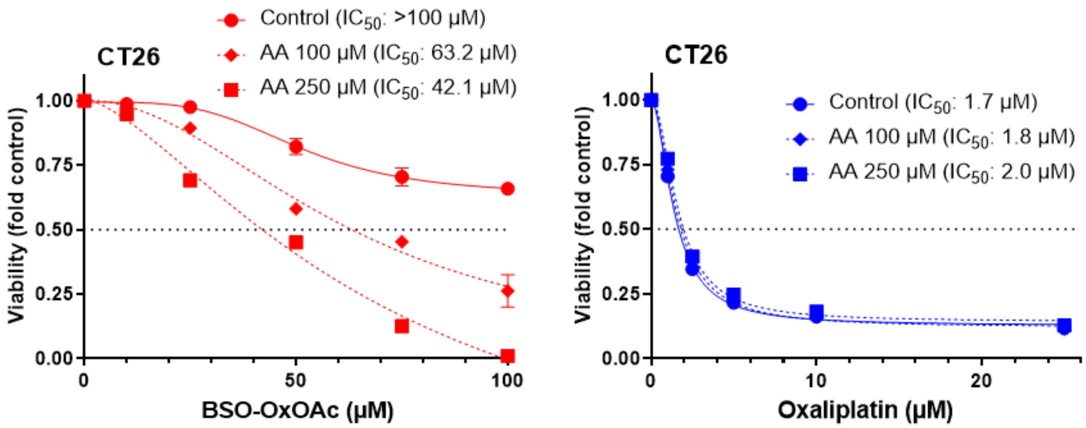

Supplementary Figure 7. Activity of **BSO-OxOAc** as compared to oxaliplatin with and without ascorbic acid (AA) on the viability of the murine colorectal cancer cell line CT26 after a 72 h continuous drug exposure. One representative experiment out of at least three performed in triplicate is shown.

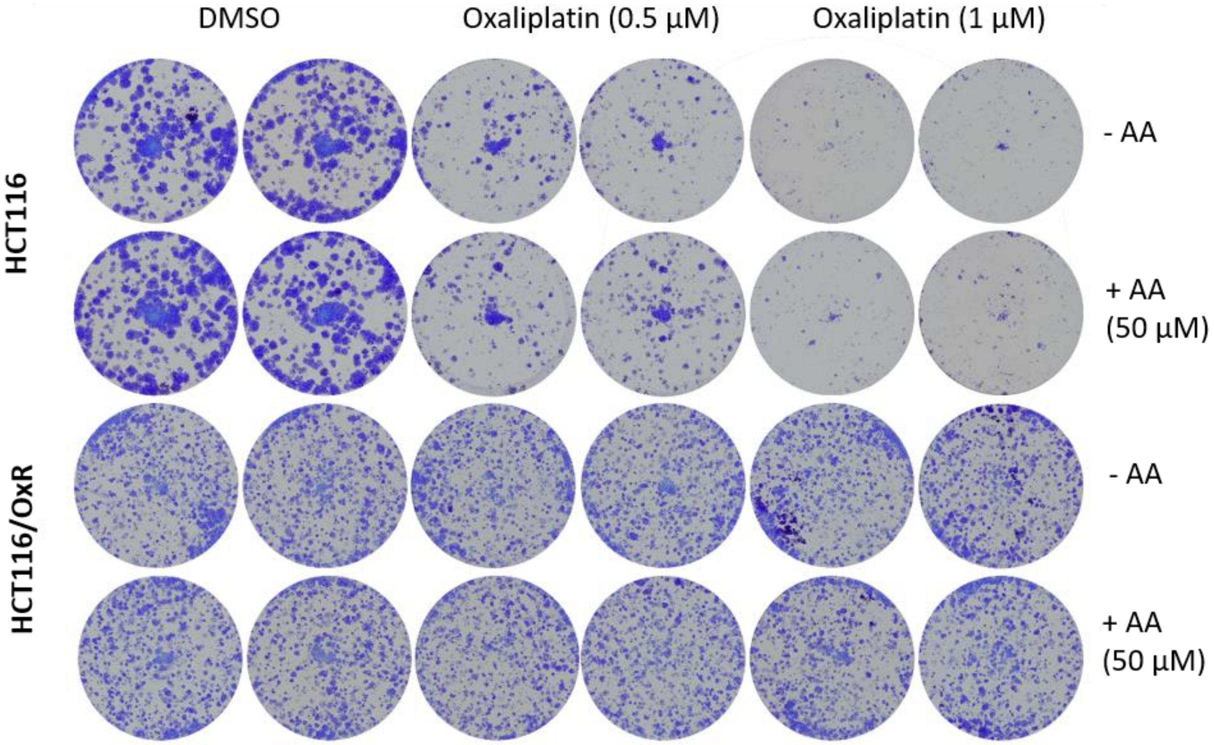

Supplementary Figure 8. Impact of oxaliplatin as single agent and in combination with the reducing agent AA (50 µM) on the clonogenic potential of HCT116 cells and the subline with acquired oxaliplatin resistance (HCT116/OxR). Sparsely seeded cells ($1x10^3$/24-well plate well) were exposed for 10 days to the indicated compounds and derived cell clones stained with crystal violet, photographed, and results evaluated by Image J software as described in Materials and Methods. One respective experiment out of three performed in duplicate is shown.

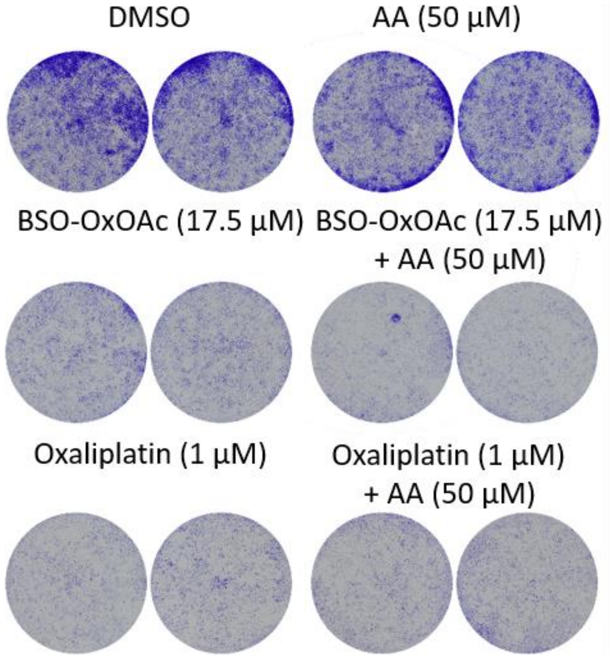

Supplementary Figure 9. Impact of **BSO-OxOAc** and oxaliplatin as single agent and in combination with AA (50 µM) on the clonogenic potential of CT26 cells. Sparsely seeded cells ($3x10^3$/24-well plate well) were exposed for 4 days to the indicated compounds and derived cell clones stained with crystal violet, photographed, and results evaluated by ImageJ software as described in Materials and Methods. One respective experiment out of three performed in duplicate is shown.

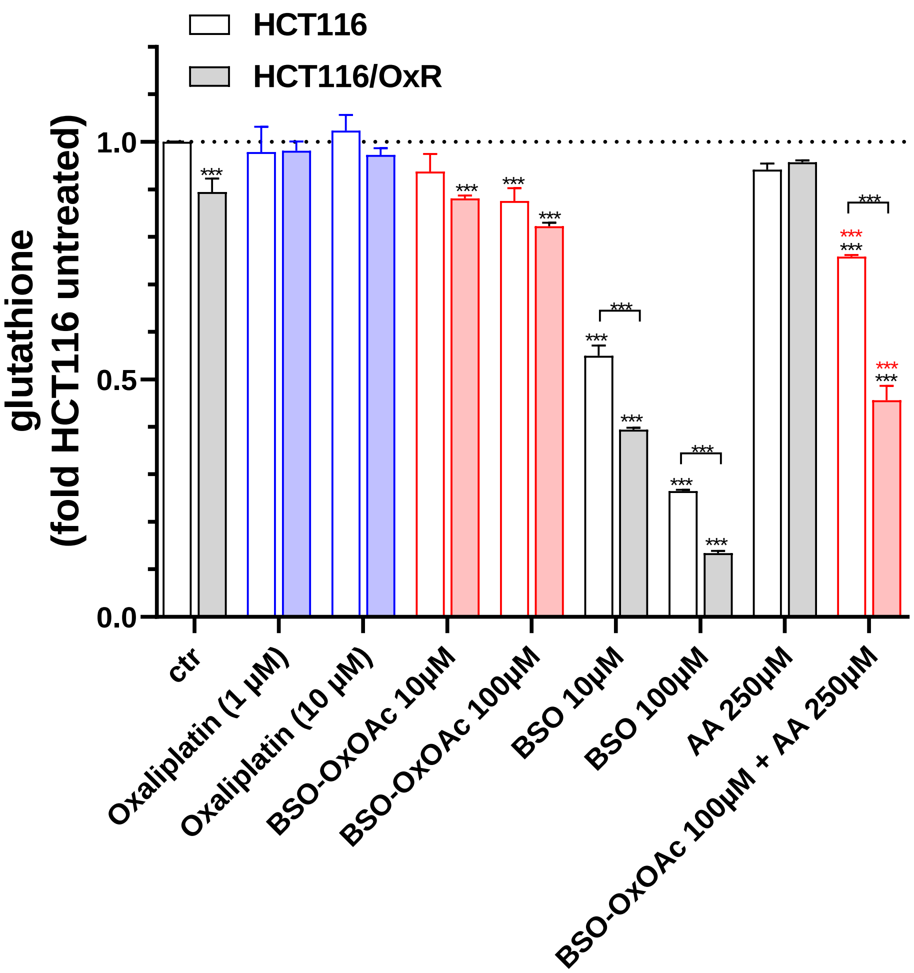

Supplementary Figure 10. Impact of the tested compounds on total glutathione (GSH) content of the HCT116 and HCT116/OxR cell model *in vitro*. Semi-confluent HCT116 and HCT116/OxR cells were exposed to the indicated drug concentrations for 24 h and the total cellular GSH content determined. Statistical significance was tested using two-way ANOVA. In all cases: ***, $p<0.001$. Significant differences of the experimental groups as compared to the DMSO control of parental HCT116 cells are indicated by black stars directly above the columns. Red stars depict the impact of the combination setting as compared to both **BSO-OxOAc** and AA as single agents. In case of a comparison between HCT116 and HCT116/OxR, only significant differences are indicated by brackets.

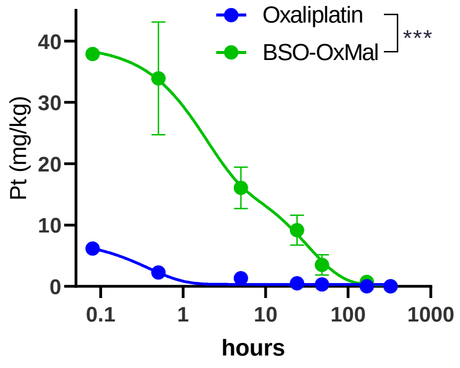

AUC

	Oxaliplatin	BSO-OxMal
Total Area	60.78	831.8
Std. Error	9.705	120.8
95% Confidence Interval	41.75 to 79.80	595.1 to 1068

Serum half-life (non linear regression, one-phase decay model)

	Oxaliplatin	BSO-OxMal
Half Life	0.2624	3.968

Serum half-life (non linear regression, two-phase decay model)

	Oxaliplatin	BSO-OxMal
Half Life (Slow)	15.38	22.28
Half Life (Fast)	0.1528	1.217

Supplementary Figure 11. Serum pharmacokinetics of **BSO-OxMal** and oxaliplatin following a single dose application at equimolar conditions as described in Materials and Methods. Platinum content was quantified by ICP-MS from serum samples of blood drawn from the facial vein at the indicated time points. The area under the concentration-time curve (AUC, total peak area; in mg $kg^{-1}h^{-1}$) and the serum half-life (h) were calculated by either a one- or two-phase nonlinear regression model using GraphPad Prism 8. Statistical significance was tested using two-way ANOVA. ***, $p<0.001$.

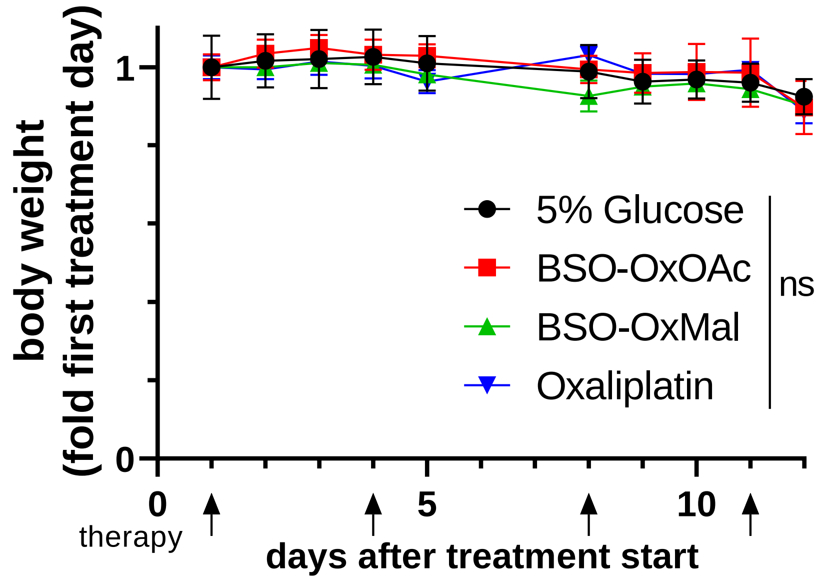

Supplementary Figure 12. Animal weights of xenograft experiments. Mice bearing CT26 allografts (n=4 per group) were treated twice a week for two weeks (black arrows) with equimolar concentrations of **BSO-OxMal** (23.5 mg/kg), **BSO-OxOAc** (19.1 mg/kg) or oxaliplatin (9 mg/kg) and mean animal weights were determined per treatment group. Statistical significance was tested using one-way ANOVA (ns: not significant).

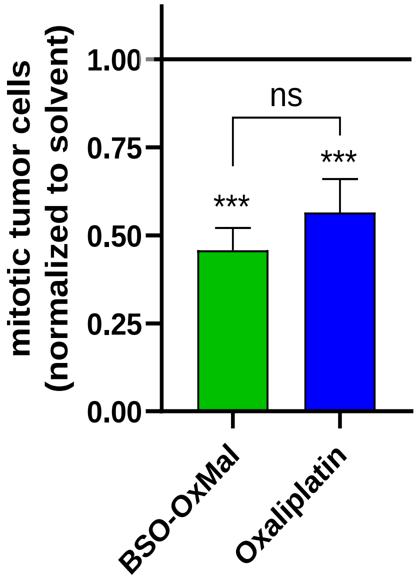

Supplementary Figure 13. Effects of **BSO-OxMal** and oxaliplatin treatment (two doses within one week) on mitotic activity of the tumor. Mice bearing CT26 allografts (n=4 per group) were treated twice a week with equimolar concentrations of **BSO-OxMal** (23.5 mg/kg) or oxaliplatin (9 mg/kg) and sacrificed 24 h after the last drug dosing. Mitotic cells were quantified by microscopically counting 6 ROI of H&E-stained sections per tumor within random non-necrotic, viable cancer regions. Statistical significance was tested using one-way ANOVA. In all cases: ***, $p<0.001$.

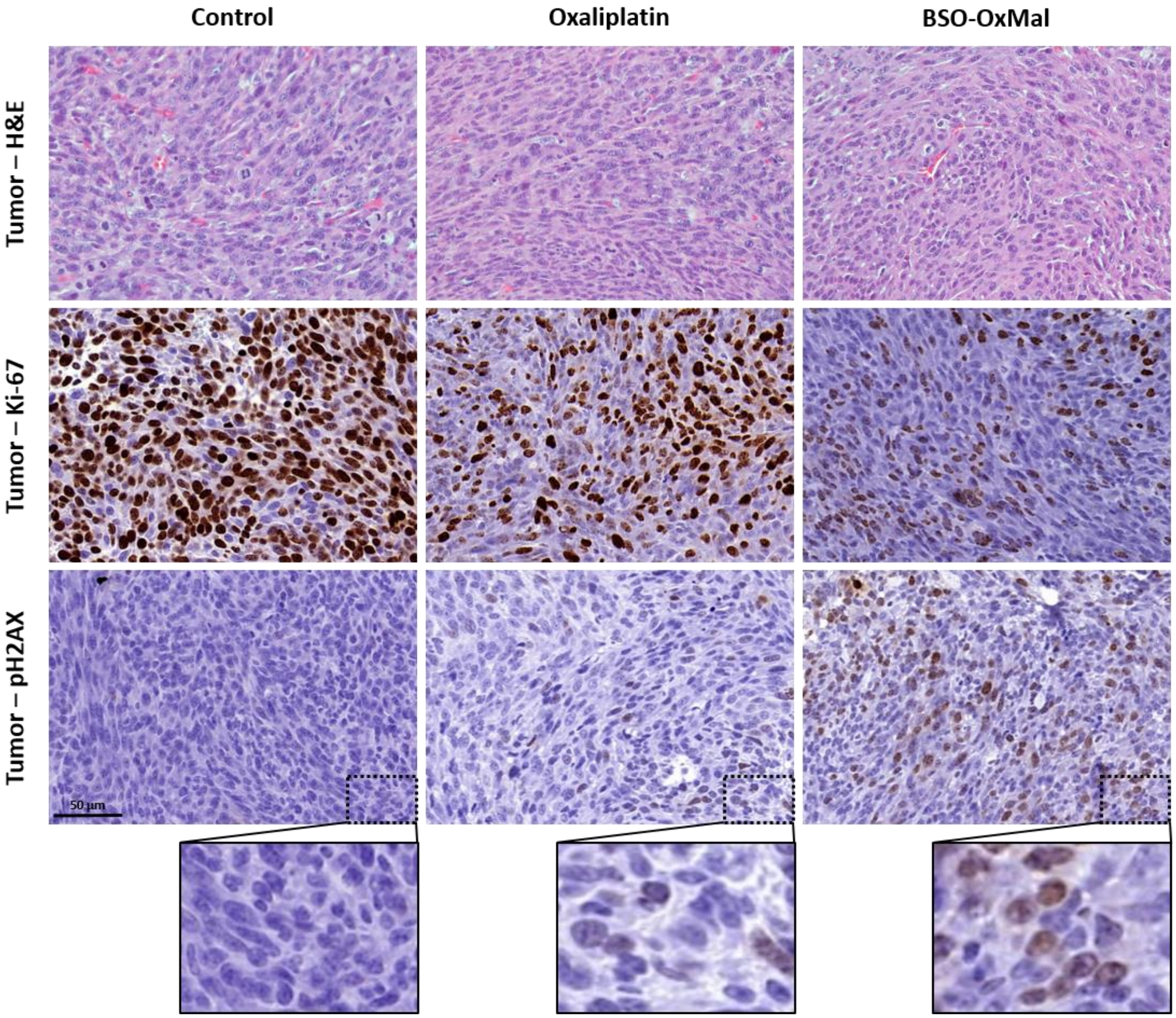

Supplementary Figure 14. Effects of **BSO-OxMal** and oxaliplatin treatment on cancer proliferative activity (Ki-67) and DNA damage (pH2AX) in CT26 tumor tissues. Mice bearing CT26 allografts (n=4 per group) were treated twice a week with solvent (control) or equimolar concentrations of **BSO-OxMal** (23.5 mg/kg) or oxaliplatin (9 mg/kg) and sacrificed 24 h after the last drug dosing. Standard H&E stainings are opposed to immunohistochemical detection of the proliferation marker Ki-67 and the DNA damage parameter pH2AX. The indicated regions in the pH2AX stained sections are shown magnified by 350%. For quantification of Ki-67 and pH2AX expression see Figure 9 of the main manuscript.

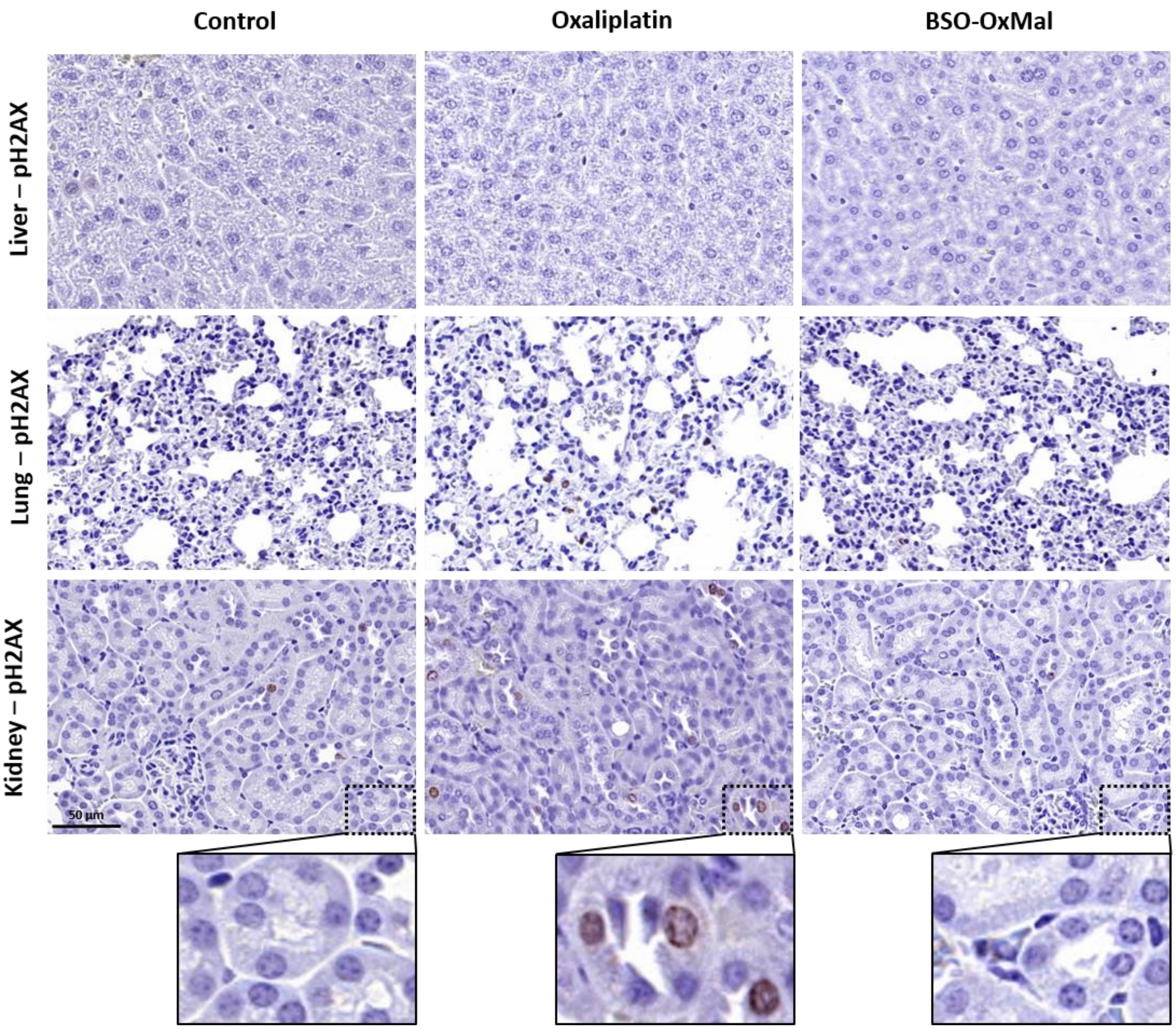

Supplementary Figure 15. Impact of **BSO-OxMal** and oxaliplatin treatment on DNA damage in healthy organs. Mice bearing CT26 allografts (n=4 per group) were treated twice a week with solvent (control) or equimolar concentrations of **BSO-OxMal** (23.5 mg/kg) or oxaliplatin (9 mg/kg) and sacrificed 24 h after the last drug dosing. The DNA damage marker pH2AX was immunohistochemically stained. Selected regions or liver, lung and kidney tissues are shown. The indicated regions in the kidney sections are shown magnified by 350%. For quantification of pH2AX in the kidney see Figure 9 of the main manuscript.
